# Supplementary figures and images for: The free fatty acid receptor GPR164 maintains intestinal homeostasis and barrier function
Source: EMBO Rep. 2025 Oct 28;26(23):5905–30. doi: 10.1038/s44319-025-00611-5 (PMC12678807; doi:10.1038/s44319-025-00611-5)

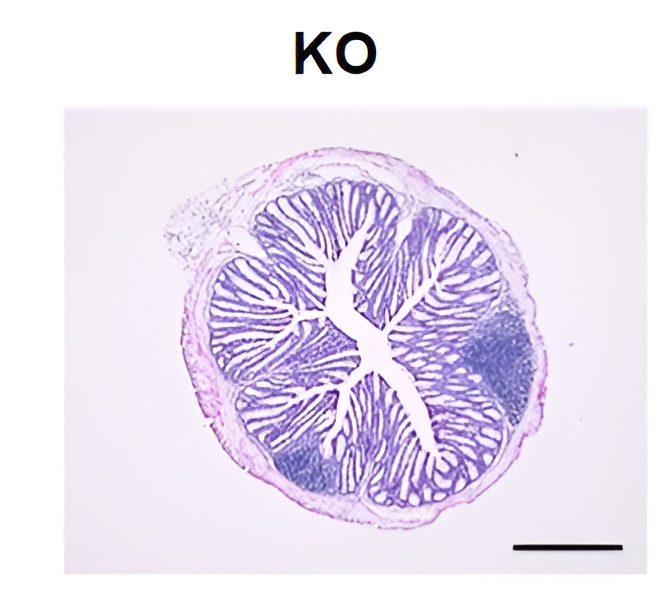

Supplement: Supplementary file 4 — Source data Fig. 2 [file 44319_2025_611_MOESM4_ESM.zip › Fig. 2/Fig. 2B_KO.jpg]

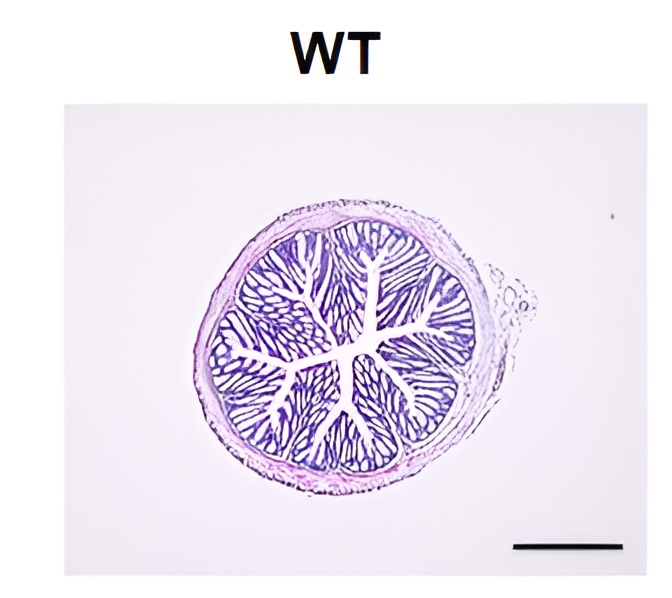

Supplement: Supplementary file 4 — Source data Fig. 2 [file 44319_2025_611_MOESM4_ESM.zip › Fig. 2/Fig. 2B_WT.jpg]

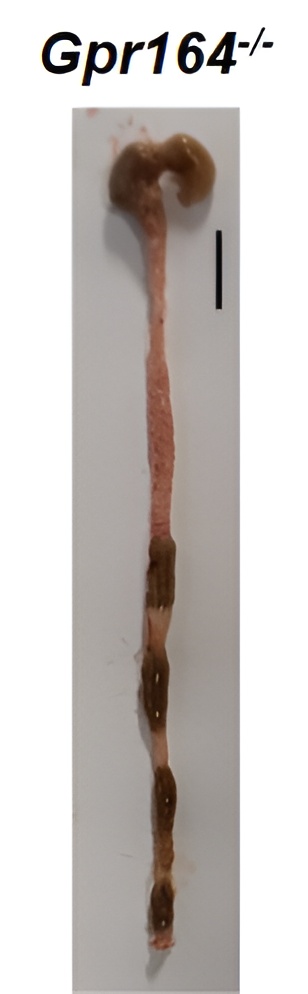

Supplement: Supplementary file 4 — Source data Fig. 2 [file 44319_2025_611_MOESM4_ESM.zip › Fig. 2/Fig. 2C_KO.jpg]

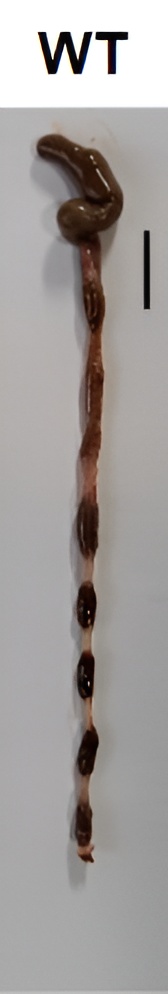

Supplement: Supplementary file 4 — Source data Fig. 2 [file 44319_2025_611_MOESM4_ESM.zip › Fig. 2/Fig. 2C_WT.jpg]

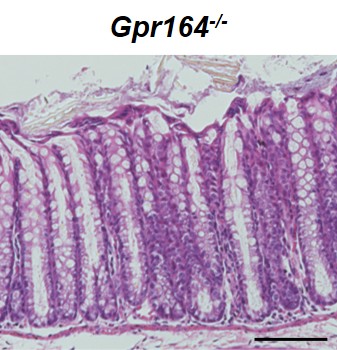

Supplement: Supplementary file 4 — Source data Fig. 2 [file 44319_2025_611_MOESM4_ESM.zip › Fig. 2/Fig. 2D_KO.jpg]

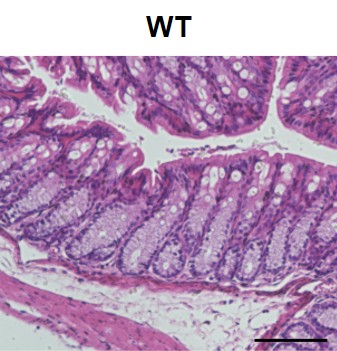

Supplement: Supplementary file 4 — Source data Fig. 2 [file 44319_2025_611_MOESM4_ESM.zip › Fig. 2/Fig. 2D_WT.jpg]

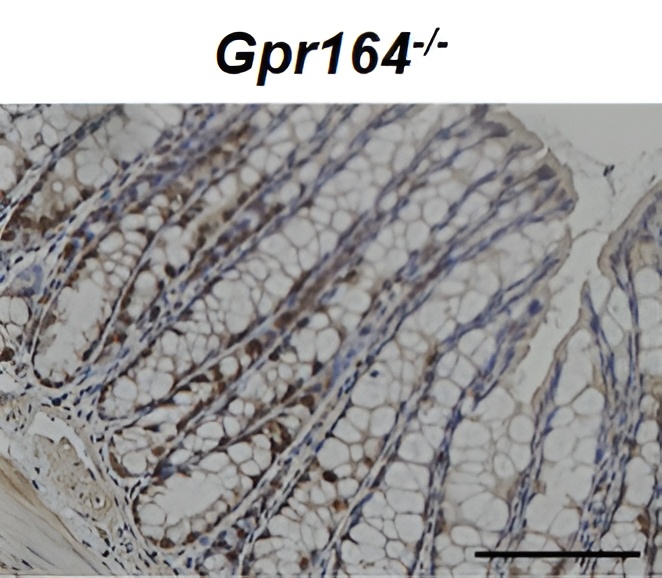

Supplement: Supplementary file 4 — Source data Fig. 2 [file 44319_2025_611_MOESM4_ESM.zip › Fig. 2/Fig. 2F_KO.jpg]

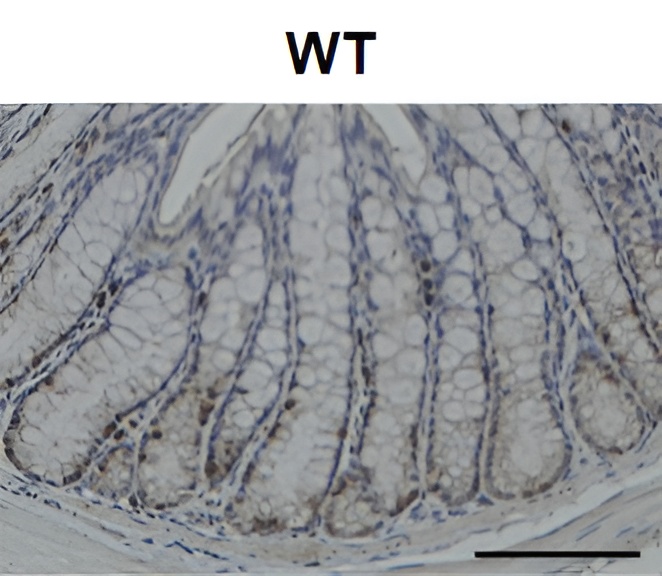

Supplement: Supplementary file 4 — Source data Fig. 2 [file 44319_2025_611_MOESM4_ESM.zip › Fig. 2/Fig. 2F_WT.jpg]

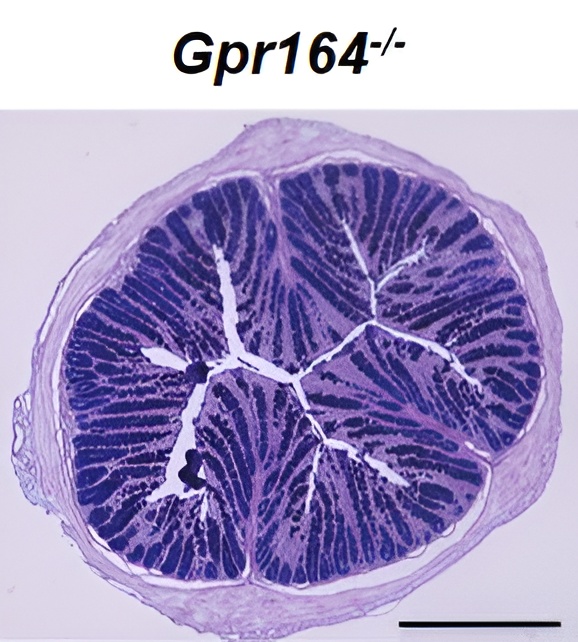

Supplement: Supplementary file 5 — Source data Fig. 3 [file 44319_2025_611_MOESM5_ESM.zip › Fig. 3/Fig. 3A_KO.jpg]

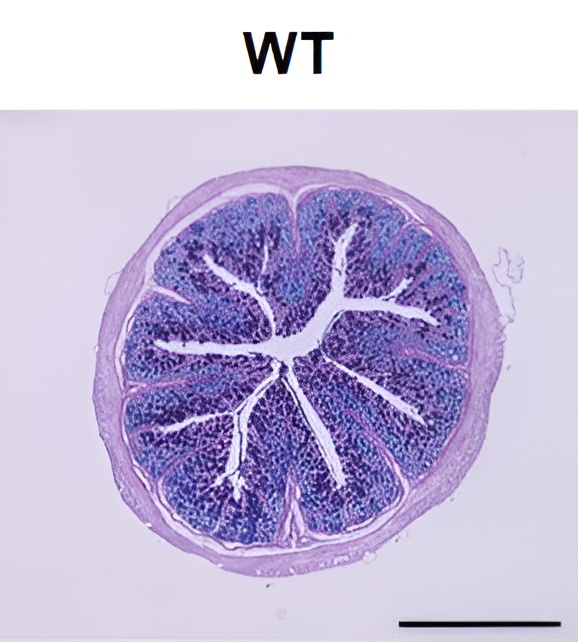

Supplement: Supplementary file 5 — Source data Fig. 3 [file 44319_2025_611_MOESM5_ESM.zip › Fig. 3/Fig. 3A_WT.jpg]

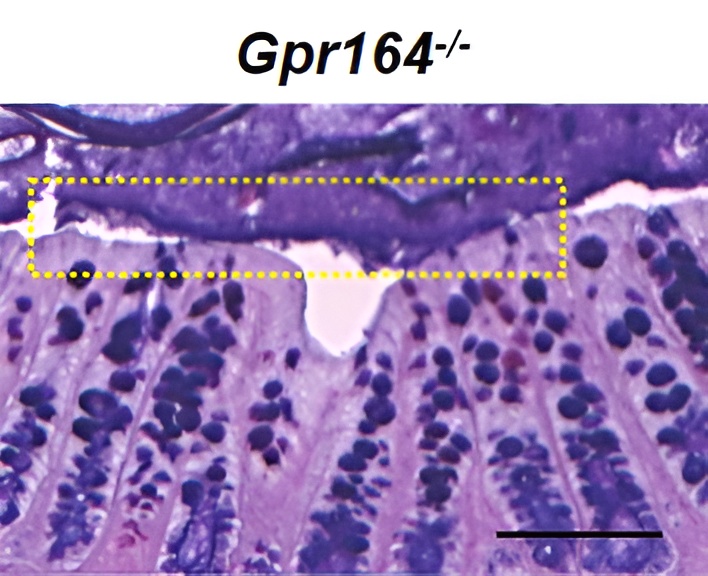

Supplement: Supplementary file 5 — Source data Fig. 3 [file 44319_2025_611_MOESM5_ESM.zip › Fig. 3/Fig. 3B_KO.jpg]

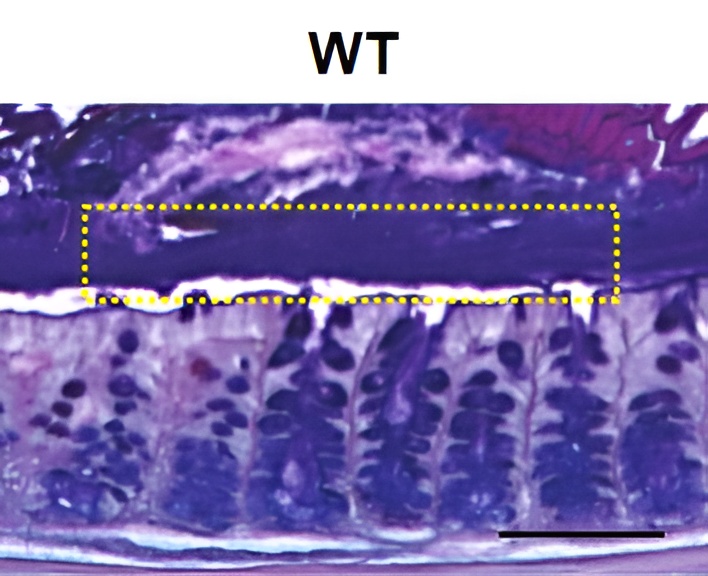

Supplement: Supplementary file 5 — Source data Fig. 3 [file 44319_2025_611_MOESM5_ESM.zip › Fig. 3/Fig. 3B_WT.jpg]

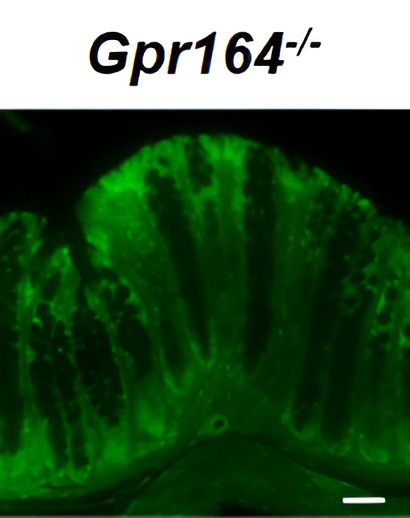

Supplement: Supplementary file 6 — Source data Fig. 4 [file 44319_2025_611_MOESM6_ESM.zip › Fig. 4/Fig. 4D_KO (b-catenin).jpg]

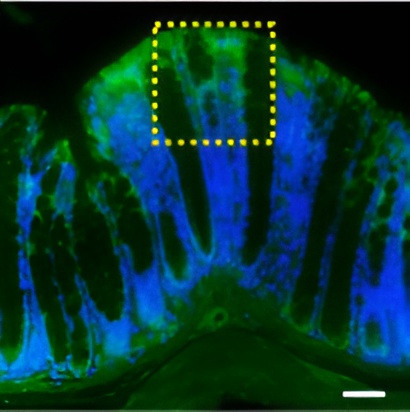

Supplement: Supplementary file 6 — Source data Fig. 4 [file 44319_2025_611_MOESM6_ESM.zip › Fig. 4/Fig. 4D_KO (b-catenin, DAPI).jpg]

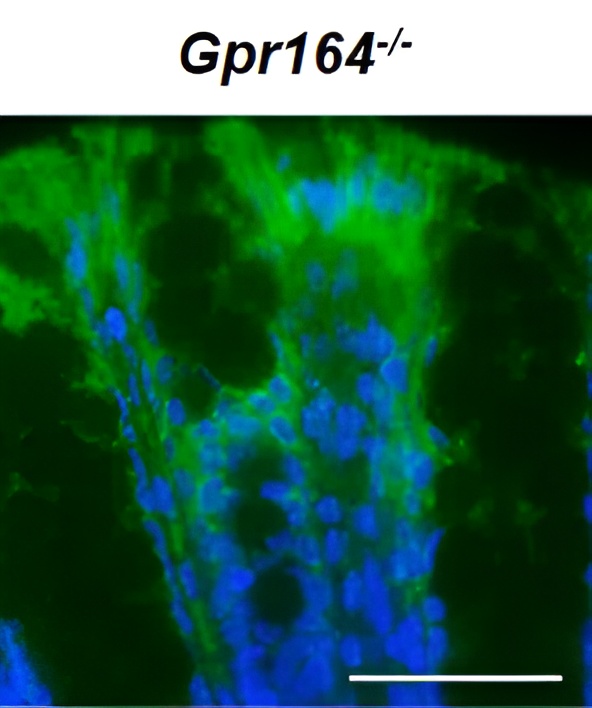

Supplement: Supplementary file 6 — Source data Fig. 4 [file 44319_2025_611_MOESM6_ESM.zip › Fig. 4/Fig. 4D_KO (High magnification).jpg]

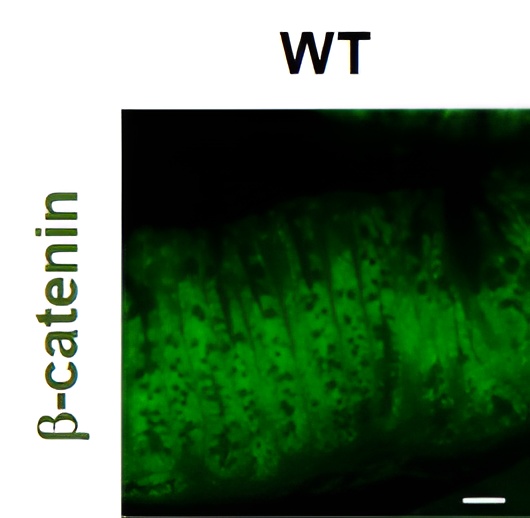

Supplement: Supplementary file 6 — Source data Fig. 4 [file 44319_2025_611_MOESM6_ESM.zip › Fig. 4/Fig. 4D_WT (b-catenin).jpg]

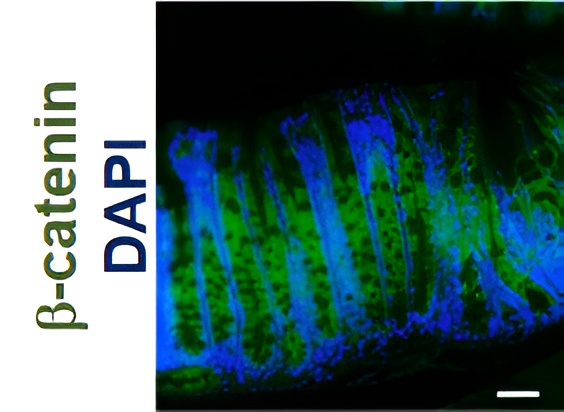

Supplement: Supplementary file 6 — Source data Fig. 4 [file 44319_2025_611_MOESM6_ESM.zip › Fig. 4/Fig. 4D_WT (b-catenin, DAPI).jpg]

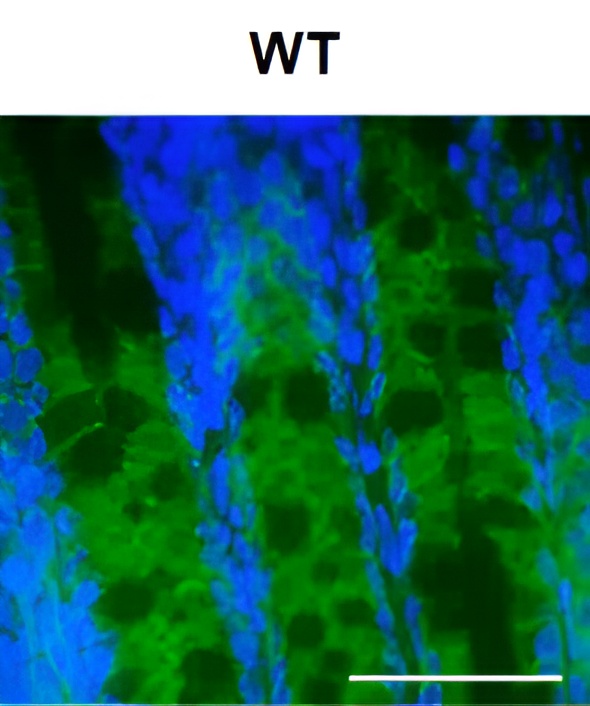

Supplement: Supplementary file 6 — Source data Fig. 4 [file 44319_2025_611_MOESM6_ESM.zip › Fig. 4/Fig. 4D_WT (High magnification).jpg]

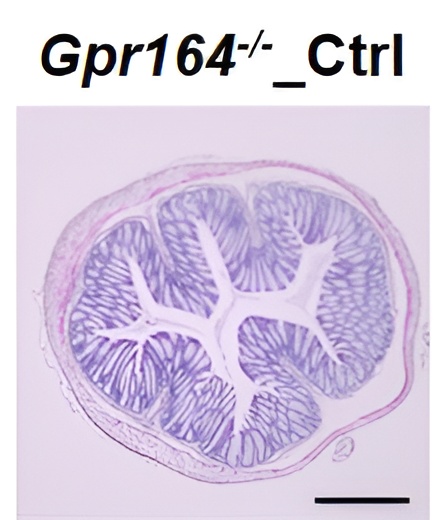

Supplement: Supplementary file 7 — Source data Fig. 5 [file 44319_2025_611_MOESM7_ESM.zip › Fig. 5/Fig. 5A_KO Ctrl.jpg]

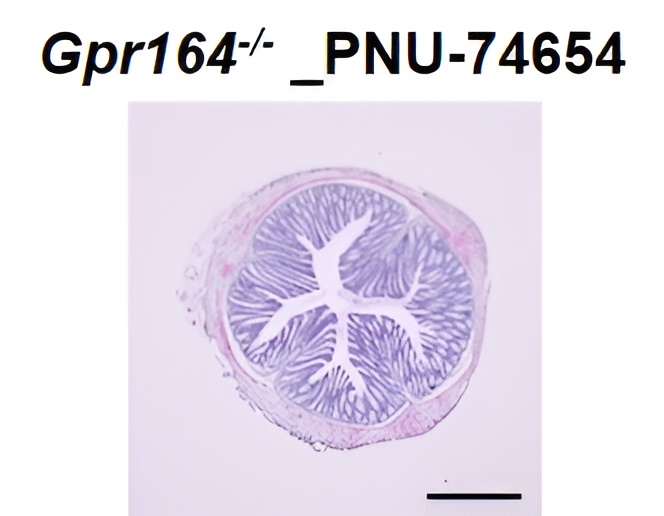

Supplement: Supplementary file 7 — Source data Fig. 5 [file 44319_2025_611_MOESM7_ESM.zip › Fig. 5/Fig. 5A_KO PNU-74654.jpg]

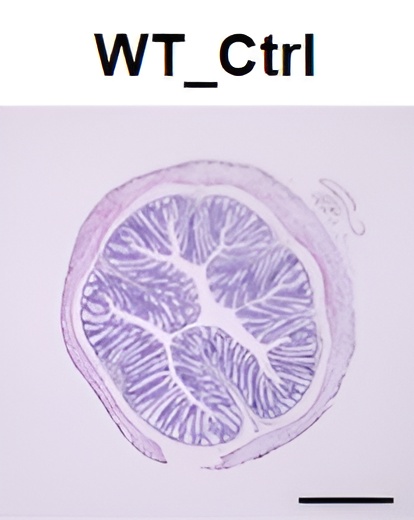

Supplement: Supplementary file 7 — Source data Fig. 5 [file 44319_2025_611_MOESM7_ESM.zip › Fig. 5/Fig. 5A_WT Ctrl.jpg]

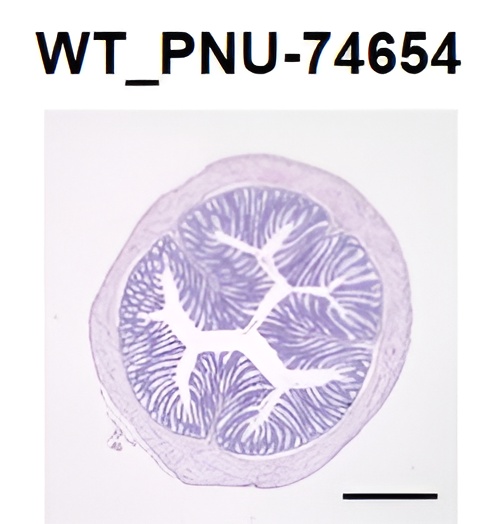

Supplement: Supplementary file 7 — Source data Fig. 5 [file 44319_2025_611_MOESM7_ESM.zip › Fig. 5/Fig. 5A_WT PNU-74654.jpg]

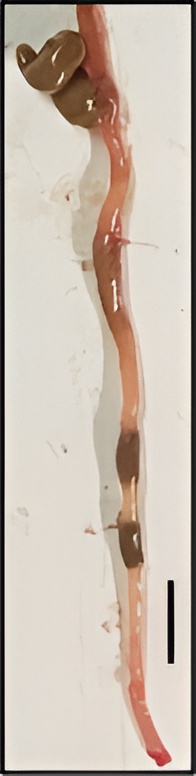

Supplement: Supplementary file 7 — Source data Fig. 5 [file 44319_2025_611_MOESM7_ESM.zip › Fig. 5/Fig. 5B_KO Ctrl.jpg]

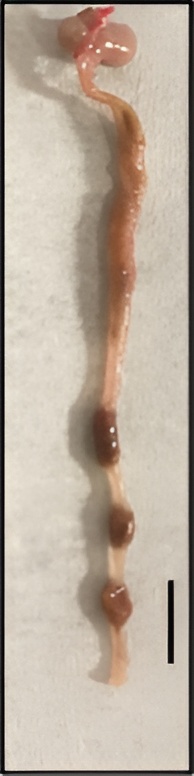

Supplement: Supplementary file 7 — Source data Fig. 5 [file 44319_2025_611_MOESM7_ESM.zip › Fig. 5/Fig. 5B_KO PNU-74654.jpg]

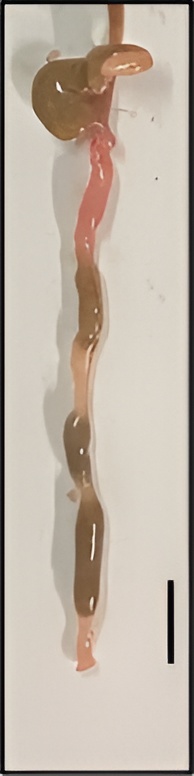

Supplement: Supplementary file 7 — Source data Fig. 5 [file 44319_2025_611_MOESM7_ESM.zip › Fig. 5/Fig. 5B_WT Ctrl.jpg]

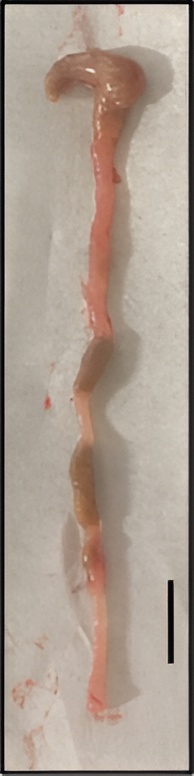

Supplement: Supplementary file 7 — Source data Fig. 5 [file 44319_2025_611_MOESM7_ESM.zip › Fig. 5/Fig. 5B_WT PNU-74654.jpg]

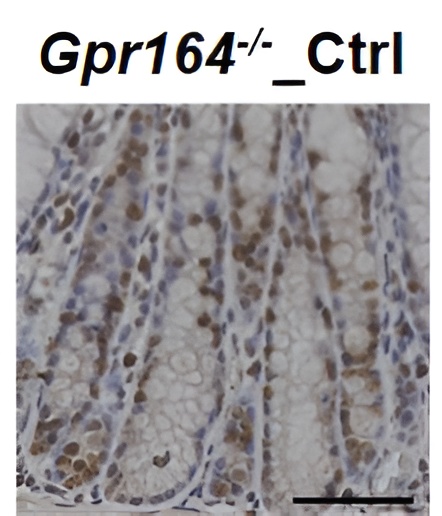

Supplement: Supplementary file 7 — Source data Fig. 5 [file 44319_2025_611_MOESM7_ESM.zip › Fig. 5/Fig. 5D_KO Ctrl.jpg]

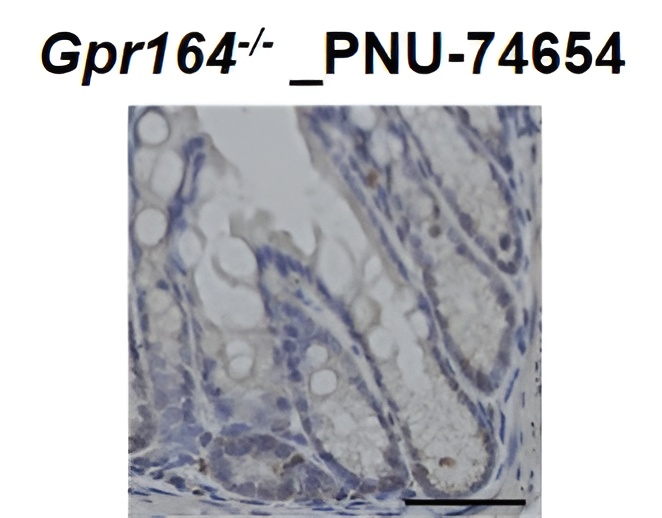

Supplement: Supplementary file 7 — Source data Fig. 5 [file 44319_2025_611_MOESM7_ESM.zip › Fig. 5/Fig. 5D_KO PNU-74654.jpg]

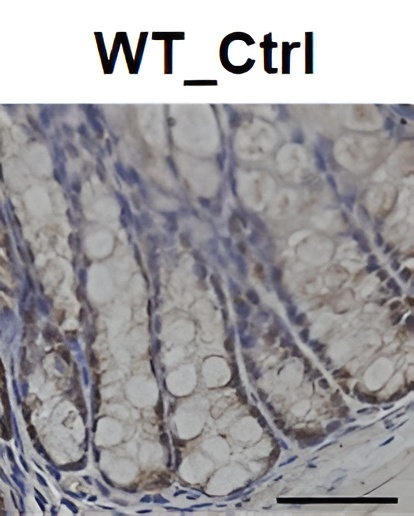

Supplement: Supplementary file 7 — Source data Fig. 5 [file 44319_2025_611_MOESM7_ESM.zip › Fig. 5/Fig. 5D_WT Ctrl.jpg]

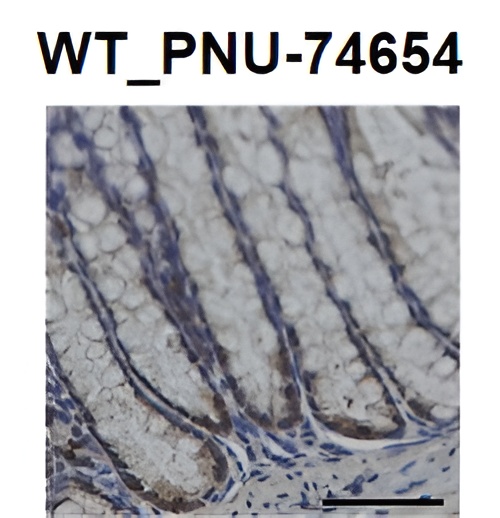

Supplement: Supplementary file 7 — Source data Fig. 5 [file 44319_2025_611_MOESM7_ESM.zip › Fig. 5/Fig. 5D_WT PNU-74654.jpg]

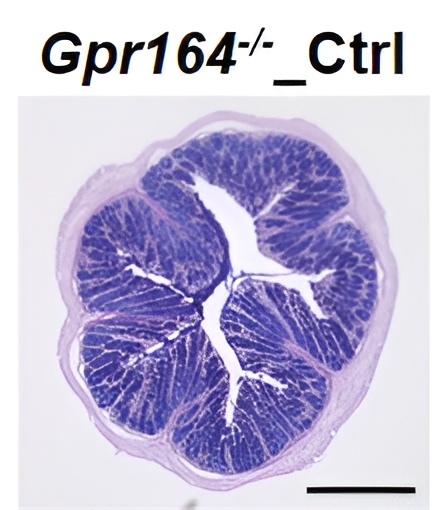

Supplement: Supplementary file 7 — Source data Fig. 5 [file 44319_2025_611_MOESM7_ESM.zip › Fig. 5/Fig. 5F_KO Ctrl.jpg]

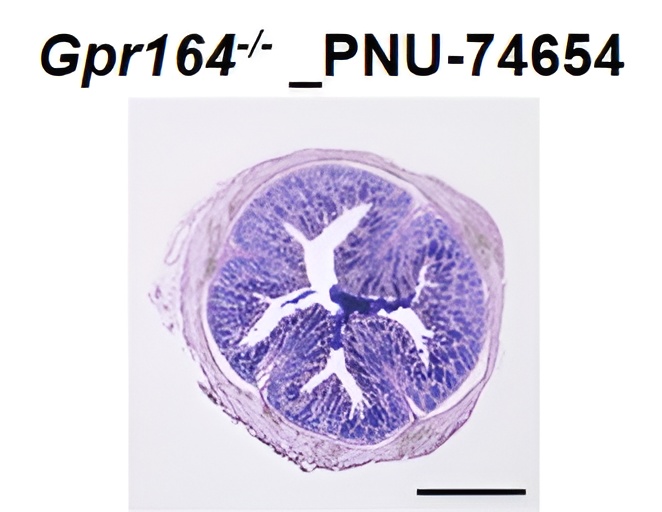

Supplement: Supplementary file 7 — Source data Fig. 5 [file 44319_2025_611_MOESM7_ESM.zip › Fig. 5/Fig. 5F_KO PNU-74654.jpg]

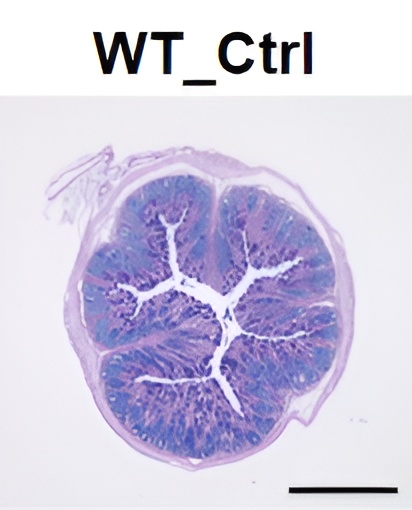

Supplement: Supplementary file 7 — Source data Fig. 5 [file 44319_2025_611_MOESM7_ESM.zip › Fig. 5/Fig. 5F_WT Ctrl.jpg]

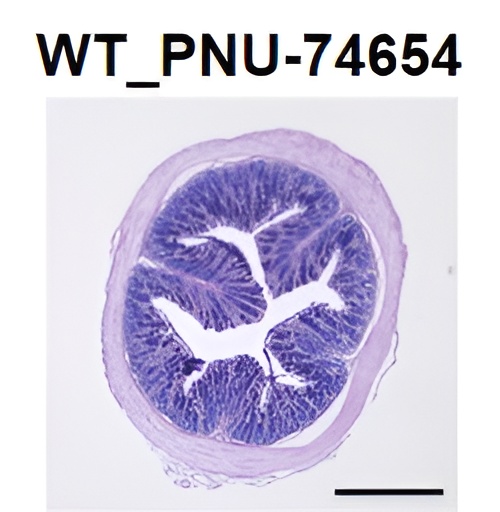

Supplement: Supplementary file 7 — Source data Fig. 5 [file 44319_2025_611_MOESM7_ESM.zip › Fig. 5/Fig. 5F_WT PNU-74654.jpg]

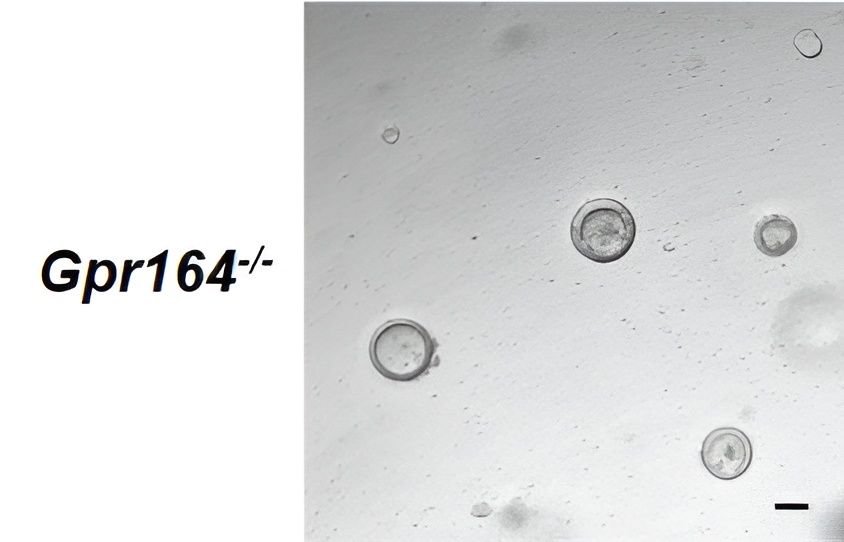

Supplement: Supplementary file 8 — Source data Fig. 6 [file 44319_2025_611_MOESM8_ESM.zip › Fig. 6/Fig. 6A_KO P0.jpg]

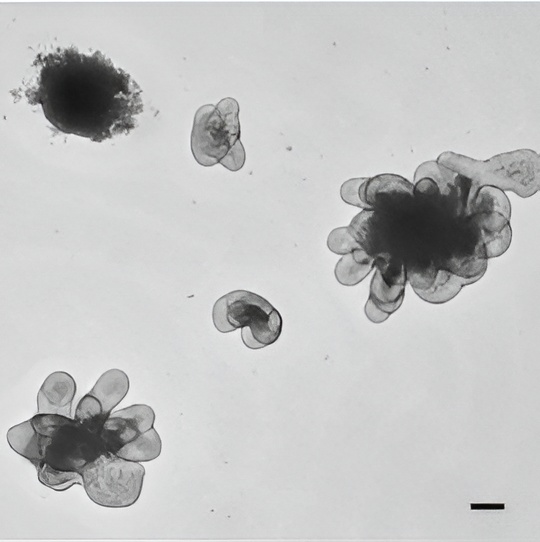

Supplement: Supplementary file 8 — Source data Fig. 6 [file 44319_2025_611_MOESM8_ESM.zip › Fig. 6/Fig. 6A_KO P1.jpg]

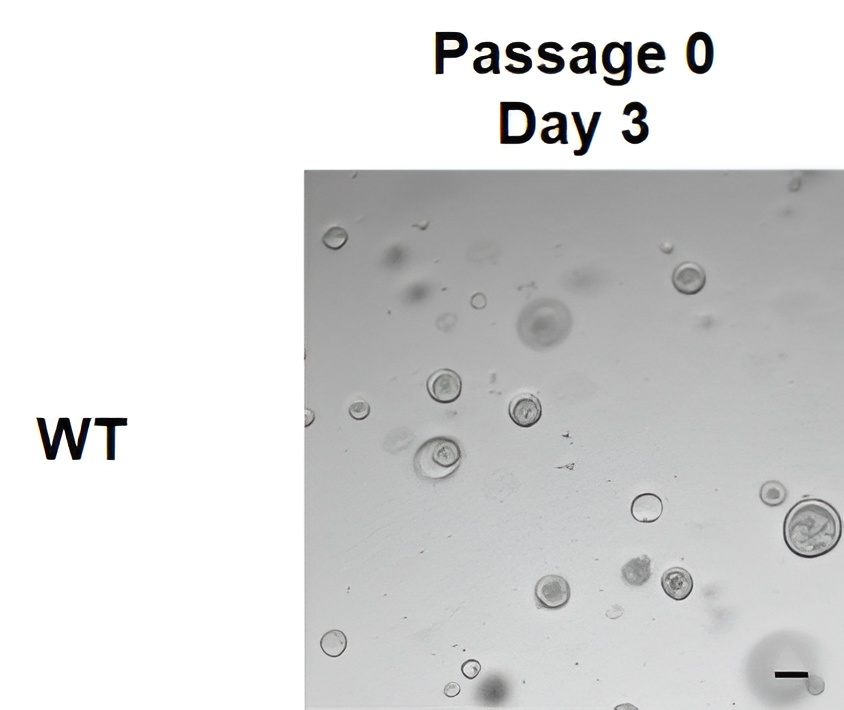

Supplement: Supplementary file 8 — Source data Fig. 6 [file 44319_2025_611_MOESM8_ESM.zip › Fig. 6/Fig. 6A_WT P0.jpg]

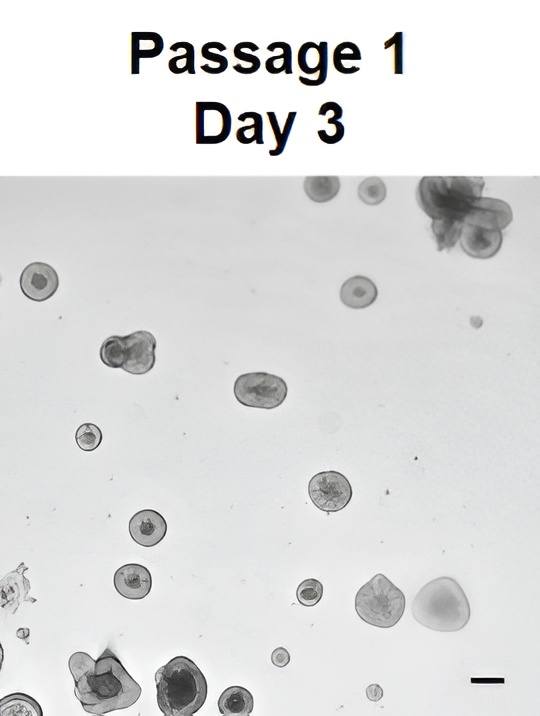

Supplement: Supplementary file 8 — Source data Fig. 6 [file 44319_2025_611_MOESM8_ESM.zip › Fig. 6/Fig. 6A_WT P1.jpg]

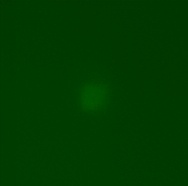

Supplement: Supplementary file 8 — Source data Fig. 6 [file 44319_2025_611_MOESM8_ESM.zip › Fig. 6/Fig. 6C_KO Butyrate (FL).jpg]

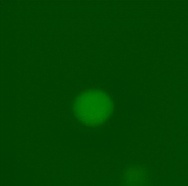

Supplement: Supplementary file 8 — Source data Fig. 6 [file 44319_2025_611_MOESM8_ESM.zip › Fig. 6/Fig. 6C_KO Butyrate Palmitate (FL).jpg]

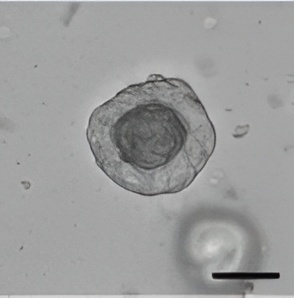

Supplement: Supplementary file 8 — Source data Fig. 6 [file 44319_2025_611_MOESM8_ESM.zip › Fig. 6/Fig. 6C_KO Butyrate Palmitate.jpg]

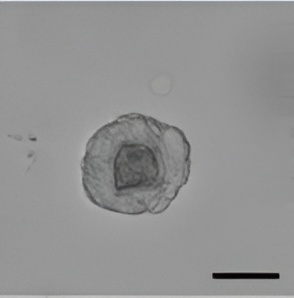

Supplement: Supplementary file 8 — Source data Fig. 6 [file 44319_2025_611_MOESM8_ESM.zip › Fig. 6/Fig. 6C_KO Butyrate.jpg]

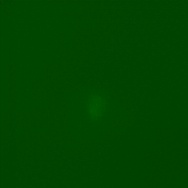

Supplement: Supplementary file 8 — Source data Fig. 6 [file 44319_2025_611_MOESM8_ESM.zip › Fig. 6/Fig. 6C_KO Ctrl (FL).jpg]

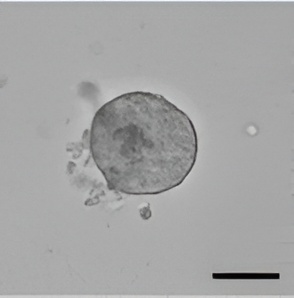

Supplement: Supplementary file 8 — Source data Fig. 6 [file 44319_2025_611_MOESM8_ESM.zip › Fig. 6/Fig. 6C_KO Ctrl.jpg]

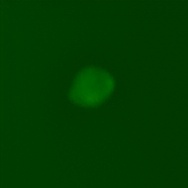

Supplement: Supplementary file 8 — Source data Fig. 6 [file 44319_2025_611_MOESM8_ESM.zip › Fig. 6/Fig. 6C_KO Palmitate (FL).jpg]

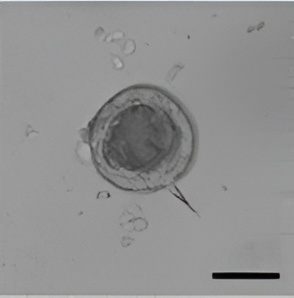

Supplement: Supplementary file 8 — Source data Fig. 6 [file 44319_2025_611_MOESM8_ESM.zip › Fig. 6/Fig. 6C_KO Palmitate.jpg]

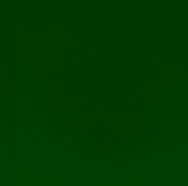

Supplement: Supplementary file 8 — Source data Fig. 6 [file 44319_2025_611_MOESM8_ESM.zip › Fig. 6/Fig. 6C_WT Butyrate (FL).jpg]

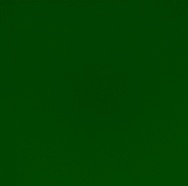

Supplement: Supplementary file 8 — Source data Fig. 6 [file 44319_2025_611_MOESM8_ESM.zip › Fig. 6/Fig. 6C_WT Butyrate Palmitate (FL).jpg]

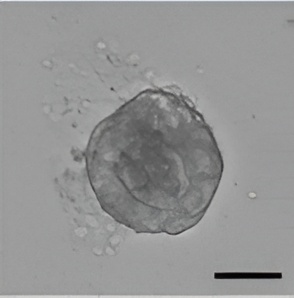

Supplement: Supplementary file 8 — Source data Fig. 6 [file 44319_2025_611_MOESM8_ESM.zip › Fig. 6/Fig. 6C_WT Butyrate Palmitate.jpg]

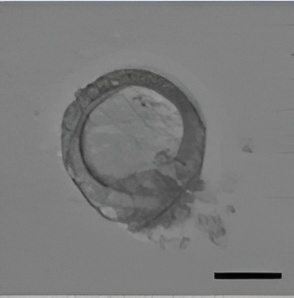

Supplement: Supplementary file 8 — Source data Fig. 6 [file 44319_2025_611_MOESM8_ESM.zip › Fig. 6/Fig. 6C_WT Butyrate.jpg]

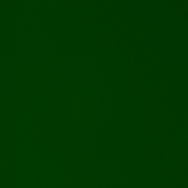

Supplement: Supplementary file 8 — Source data Fig. 6 [file 44319_2025_611_MOESM8_ESM.zip › Fig. 6/Fig. 6C_WT Ctrl (FL).jpg]

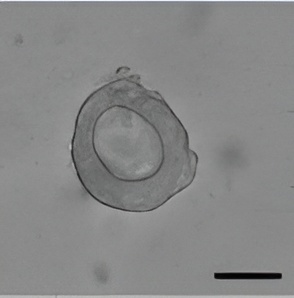

Supplement: Supplementary file 8 — Source data Fig. 6 [file 44319_2025_611_MOESM8_ESM.zip › Fig. 6/Fig. 6C_WT Ctrl.jpg]

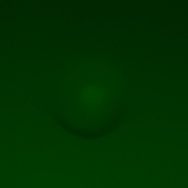

Supplement: Supplementary file 8 — Source data Fig. 6 [file 44319_2025_611_MOESM8_ESM.zip › Fig. 6/Fig. 6C_WT Palmitate (FL).jpg]

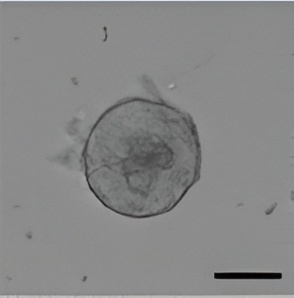

Supplement: Supplementary file 8 — Source data Fig. 6 [file 44319_2025_611_MOESM8_ESM.zip › Fig. 6/Fig. 6C_WT Palmitate.jpg]

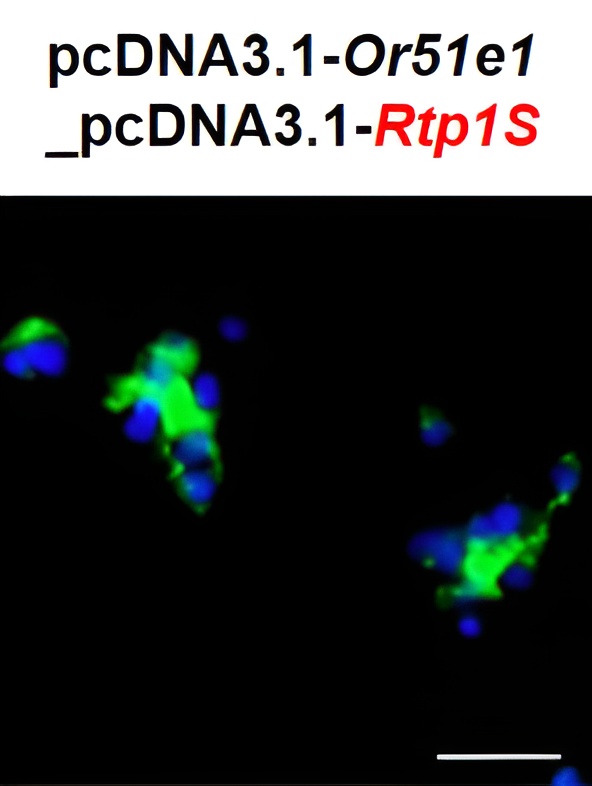

Supplement: Supplementary file 9 — Figure EV1 Source Data [file 44319_2025_611_MOESM9_ESM.zip › Figure EV1/Fig. EV1A/Fig. EV1A_RTP1S.jpg]

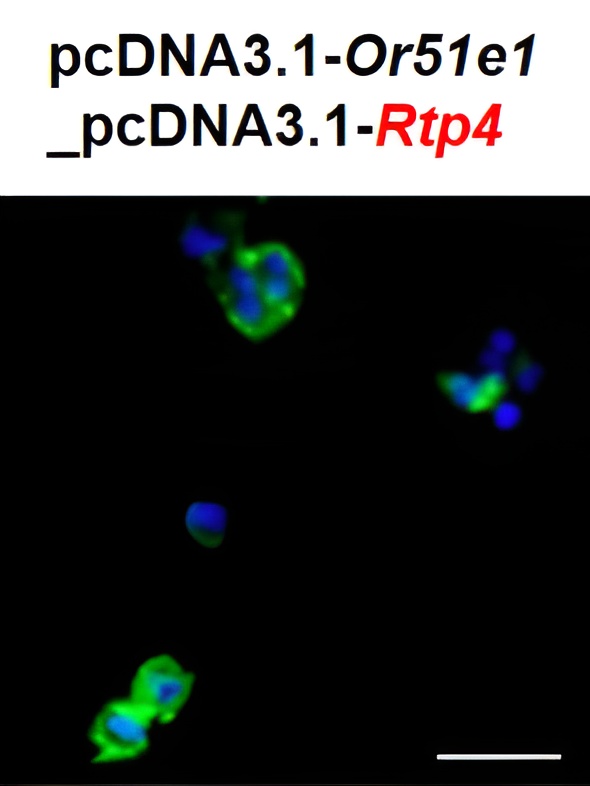

Supplement: Supplementary file 9 — Figure EV1 Source Data [file 44319_2025_611_MOESM9_ESM.zip › Figure EV1/Fig. EV1A/Fig. EV1A_RTP4.jpg]

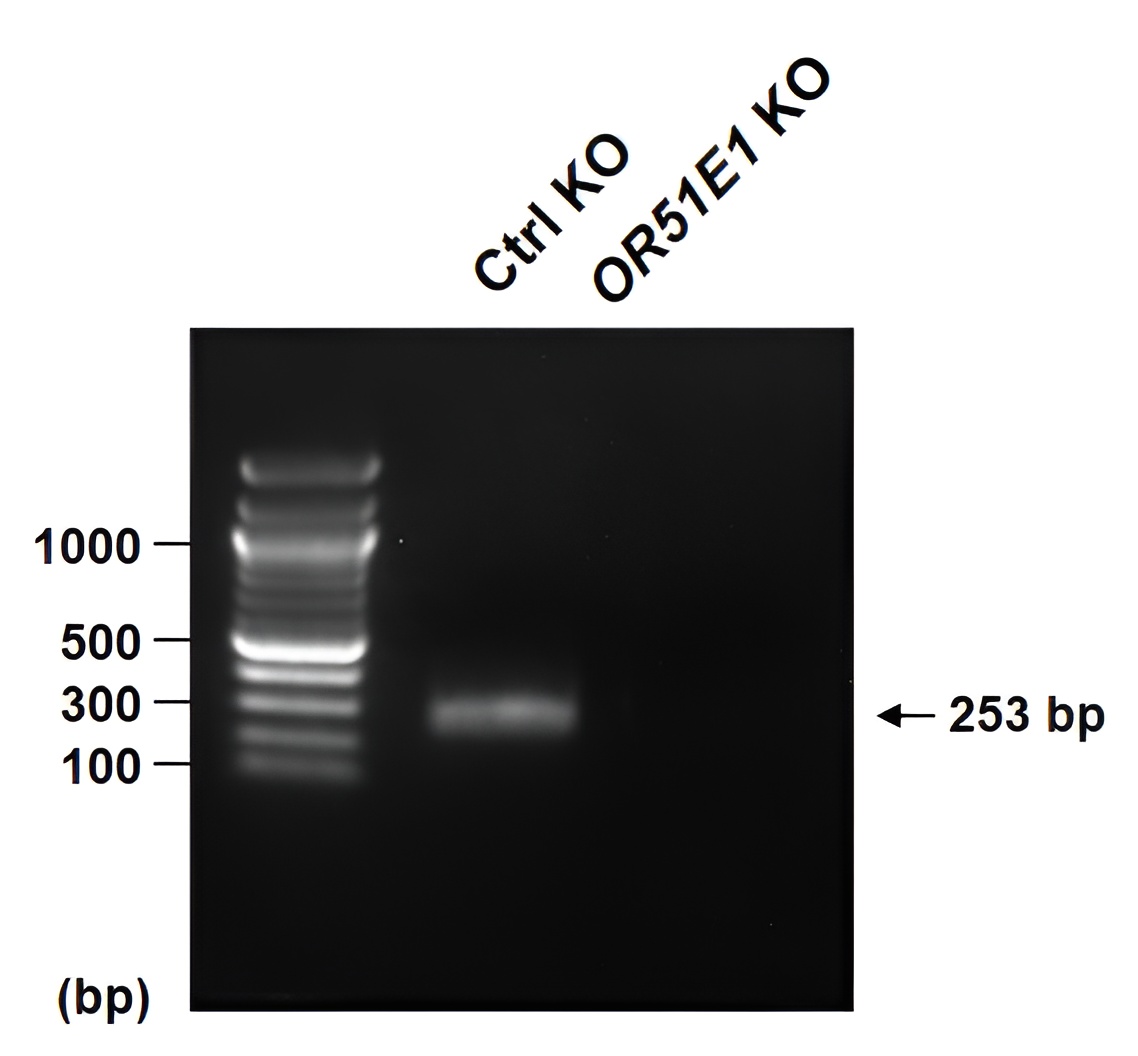

Supplement: Supplementary file 9 — Figure EV1 Source Data [file 44319_2025_611_MOESM9_ESM.zip › Figure EV1/Fig. EV1B/Fig. EV1B (left).jpg]

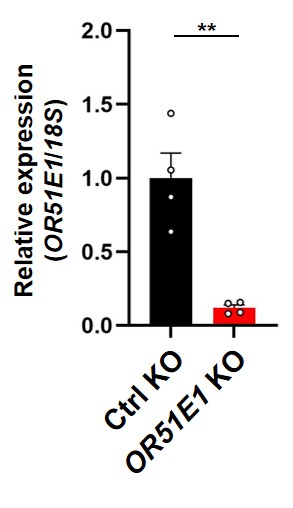

Supplement: Supplementary file 9 — Figure EV1 Source Data [file 44319_2025_611_MOESM9_ESM.zip › Figure EV1/Fig. EV1B/Fig. EV1B (right).jpg]

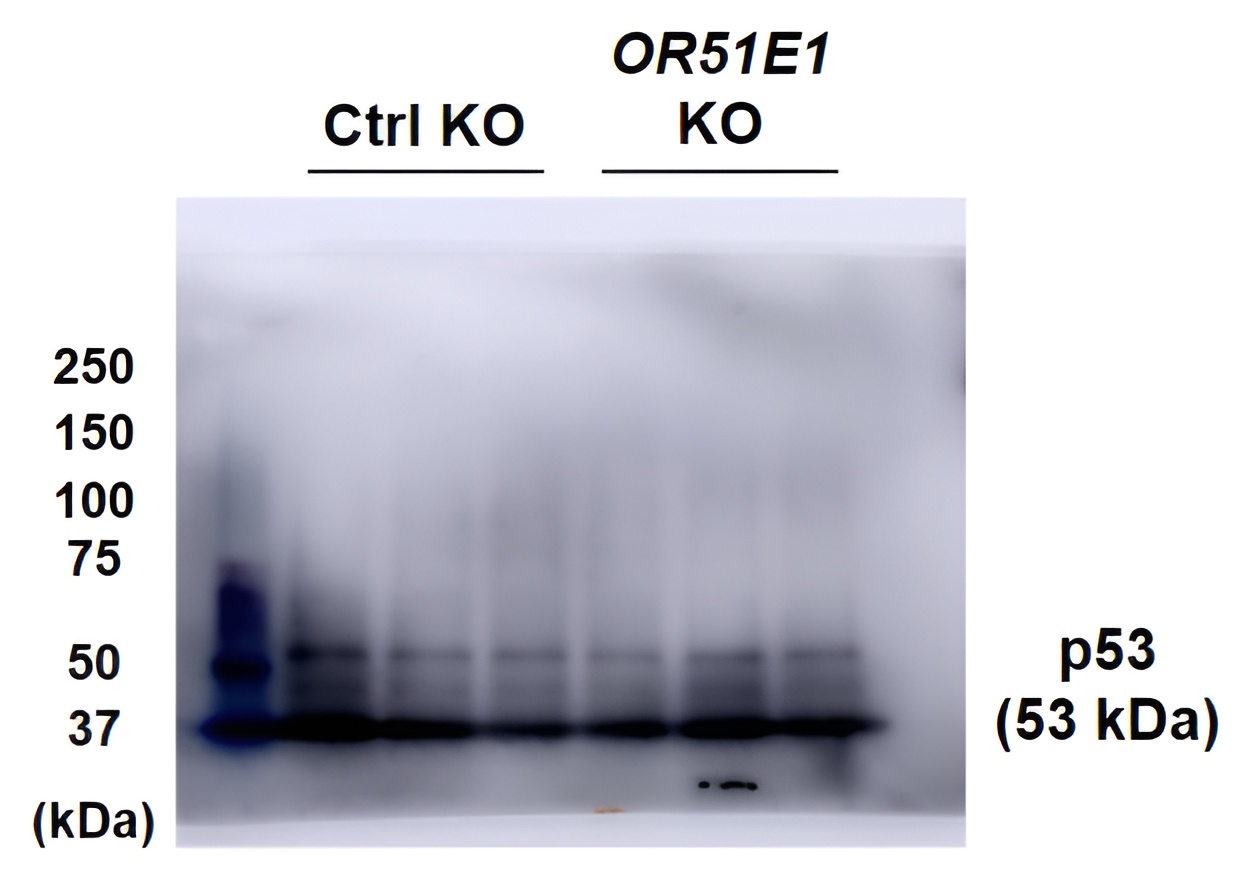

Supplement: Supplementary file 9 — Figure EV1 Source Data [file 44319_2025_611_MOESM9_ESM.zip › Figure EV1/Fig. EV1C/Fig. EV1C p53.jpg]

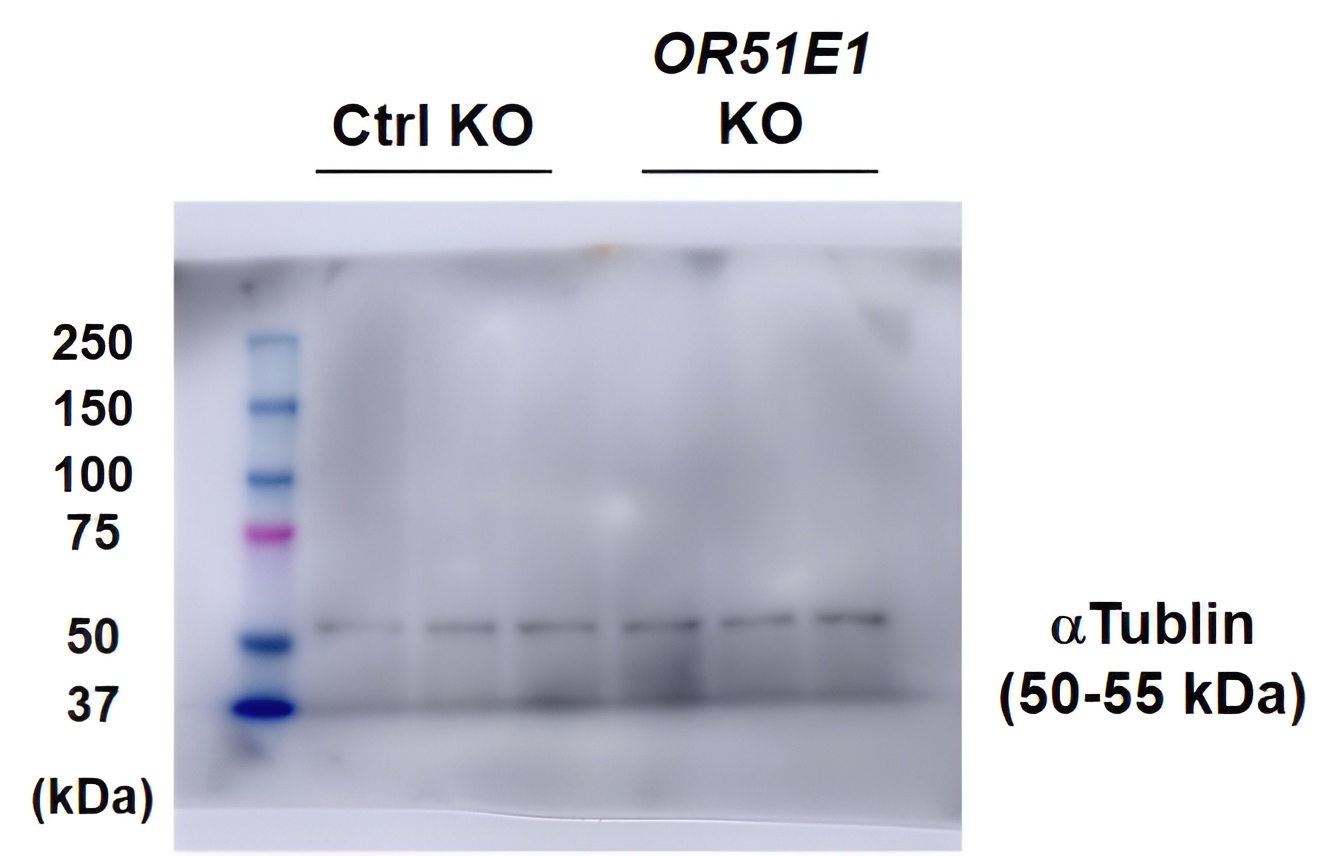

Supplement: Supplementary file 9 — Figure EV1 Source Data [file 44319_2025_611_MOESM9_ESM.zip › Figure EV1/Fig. EV1C/Fig. EV1C tublin.jpg]

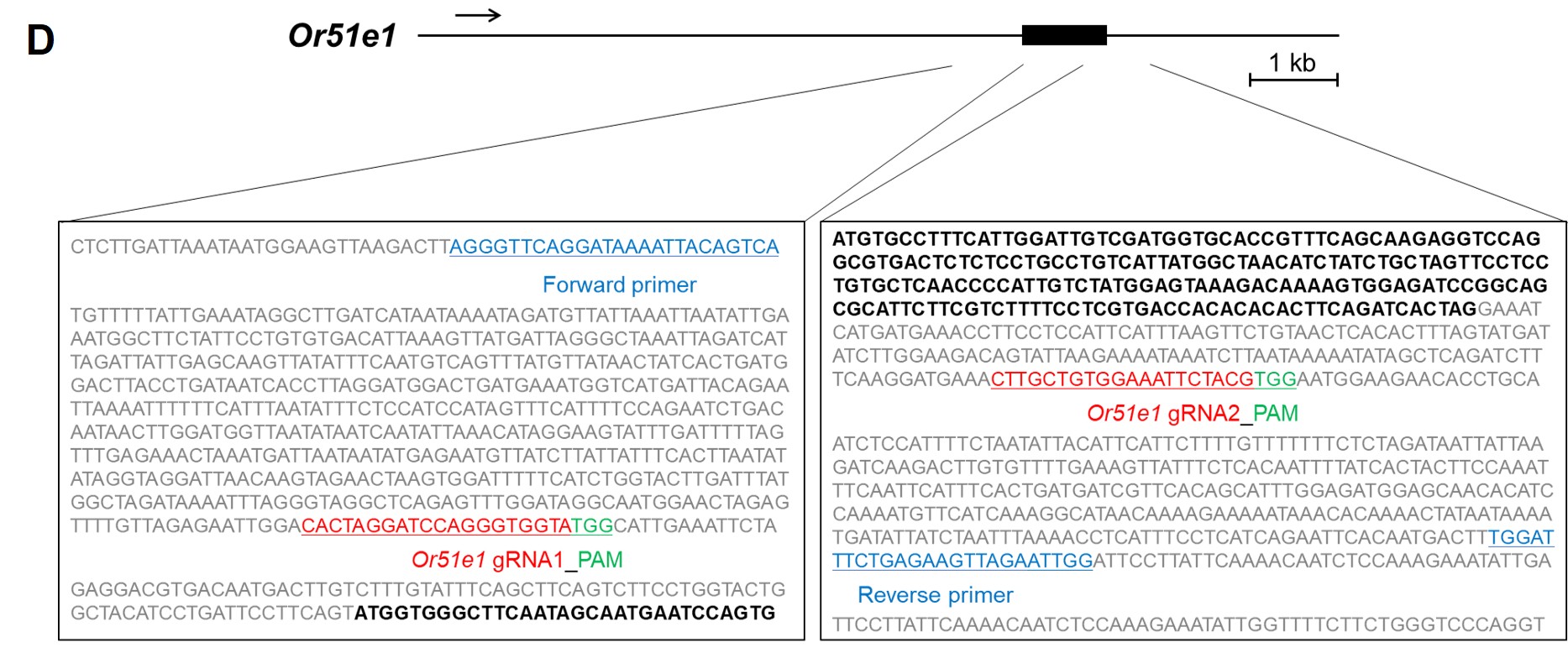

Supplement: Supplementary file 9 — Figure EV1 Source Data [file 44319_2025_611_MOESM9_ESM.zip › Figure EV1/Fig. EV1D/Fig. EV1D.jpg]

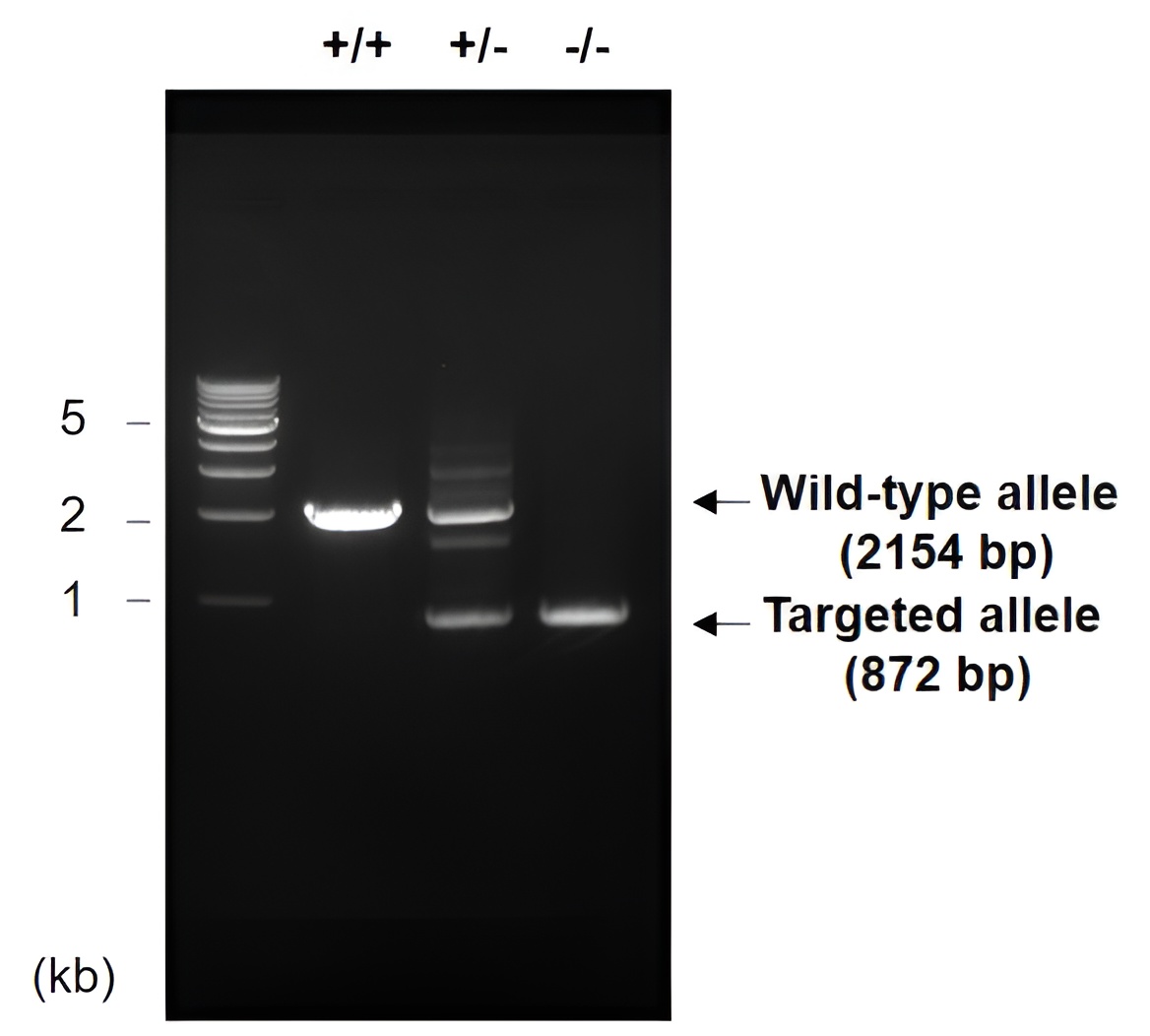

Supplement: Supplementary file 9 — Figure EV1 Source Data [file 44319_2025_611_MOESM9_ESM.zip › Figure EV1/Fig. EV1E/Fig. EV1E.jpg]

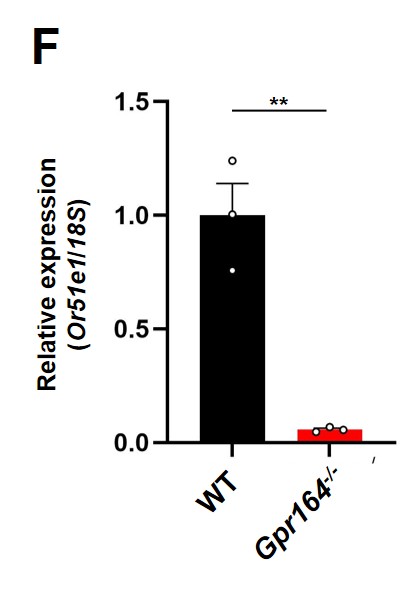

Supplement: Supplementary file 9 — Figure EV1 Source Data [file 44319_2025_611_MOESM9_ESM.zip › Figure EV1/Fig. EV1F/Fig. EV1F.jpg]

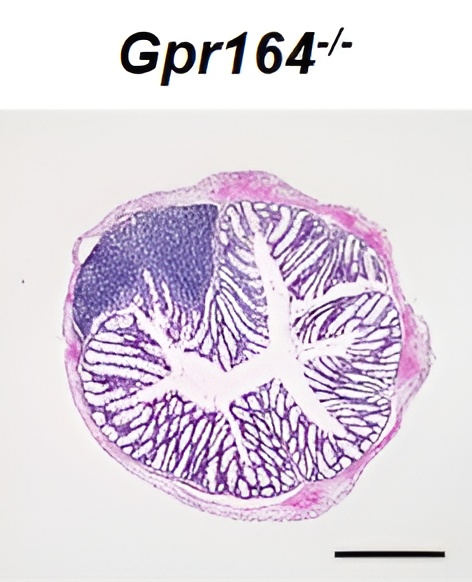

Supplement: Supplementary file 10 — Figure EV2 Source Data [file 44319_2025_611_MOESM10_ESM.zip › Figure EV2/Fig. EV2A/Fig. EV2A KO.jpg]

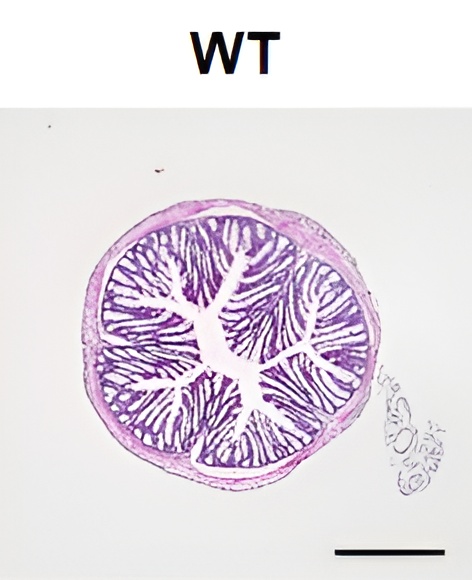

Supplement: Supplementary file 10 — Figure EV2 Source Data [file 44319_2025_611_MOESM10_ESM.zip › Figure EV2/Fig. EV2A/Fig. EV2A WT.jpg]

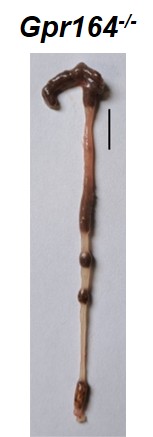

Supplement: Supplementary file 10 — Figure EV2 Source Data [file 44319_2025_611_MOESM10_ESM.zip › Figure EV2/Fig. EV2B/Fig. EV2B KO.jpg]

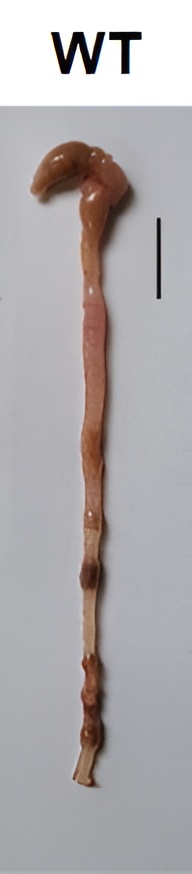

Supplement: Supplementary file 10 — Figure EV2 Source Data [file 44319_2025_611_MOESM10_ESM.zip › Figure EV2/Fig. EV2B/Fig. EV2B WT.jpg]

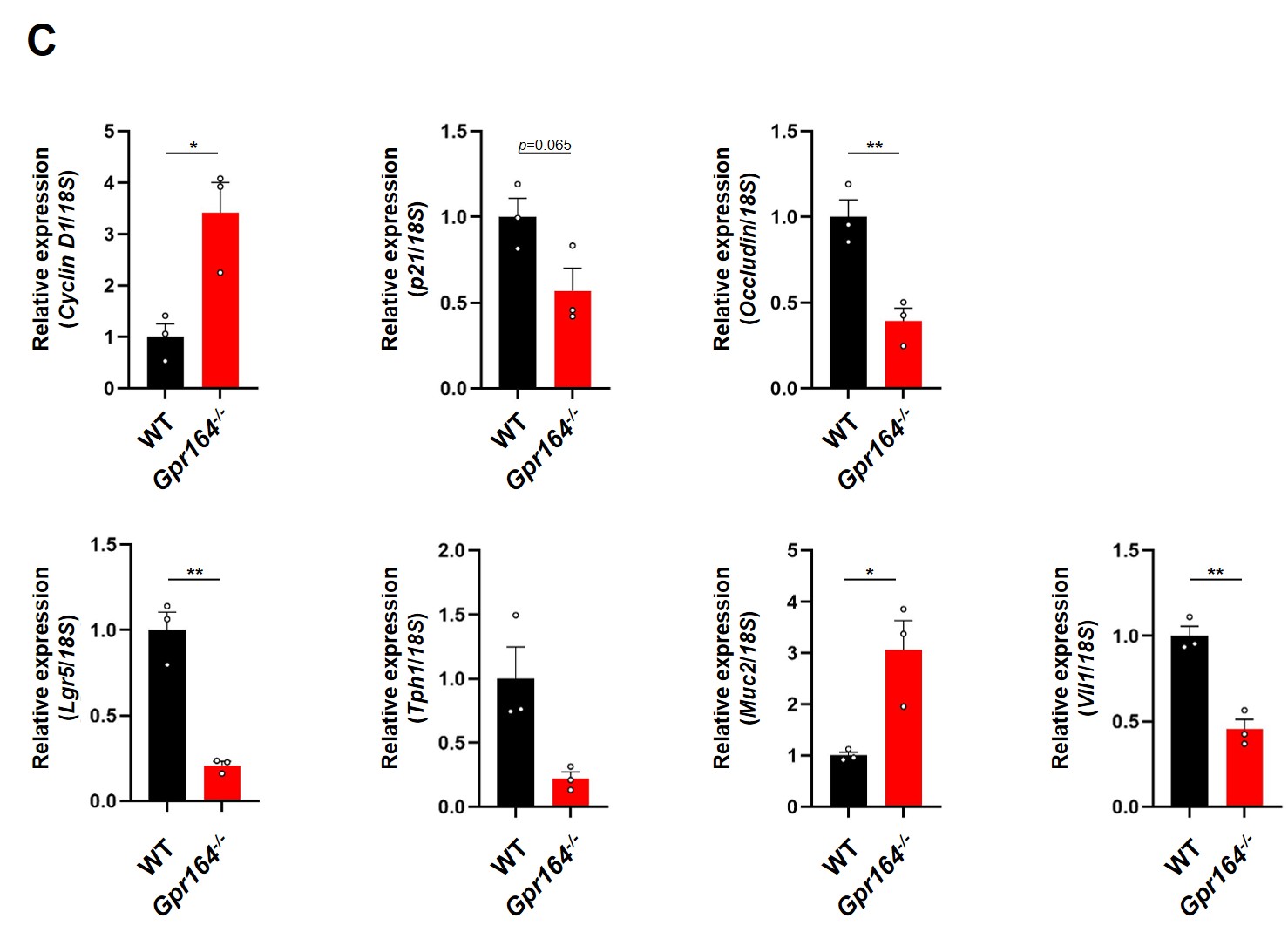

Supplement: Supplementary file 10 — Figure EV2 Source Data [file 44319_2025_611_MOESM10_ESM.zip › Figure EV2/Fig. EV2C/Fig. EV2C.jpg]

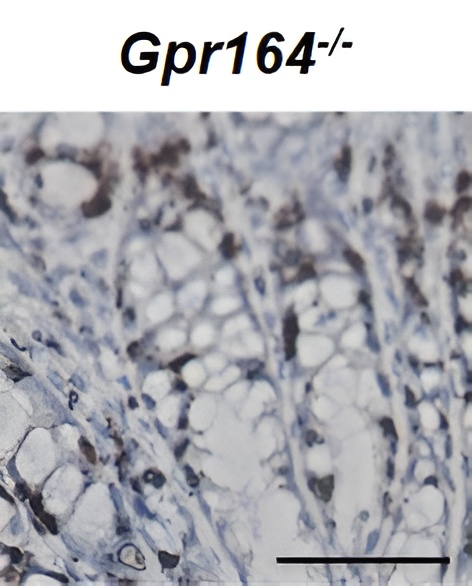

Supplement: Supplementary file 11 — Figure EV3 Source Data [file 44319_2025_611_MOESM11_ESM.zip › Figure EV3/Fig. EV3A/Fig. EV3A KO.jpg]

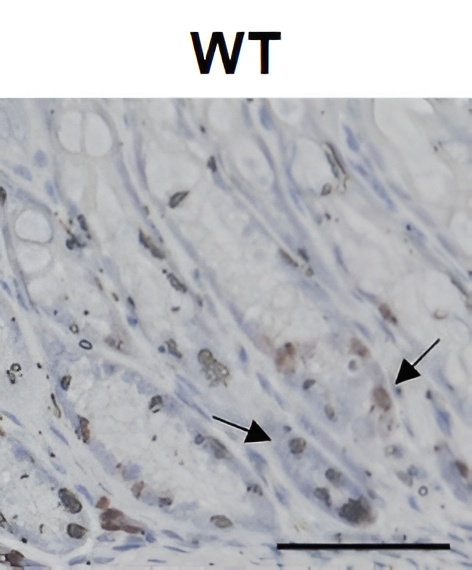

Supplement: Supplementary file 11 — Figure EV3 Source Data [file 44319_2025_611_MOESM11_ESM.zip › Figure EV3/Fig. EV3A/Fig. EV3A WT.jpg]

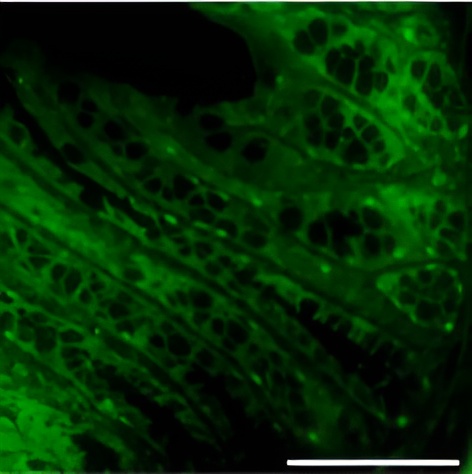

Supplement: Supplementary file 11 — Figure EV3 Source Data [file 44319_2025_611_MOESM11_ESM.zip › Figure EV3/Fig. EV3B/Fig. EV3B KO (cleaved caspase-3).jpg]

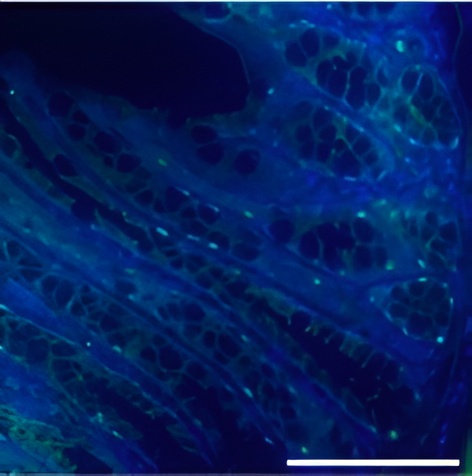

Supplement: Supplementary file 11 — Figure EV3 Source Data [file 44319_2025_611_MOESM11_ESM.zip › Figure EV3/Fig. EV3B/Fig. EV3B KO (cleaved caspase-3_DAPI).jpg]

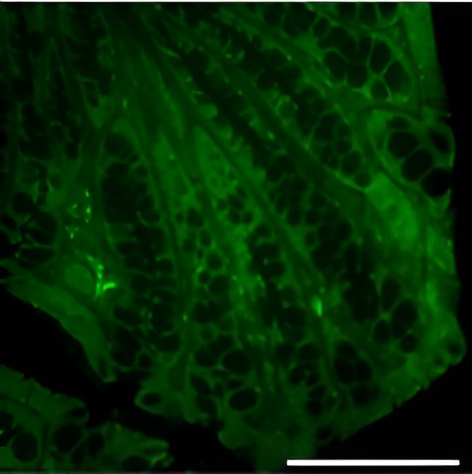

Supplement: Supplementary file 11 — Figure EV3 Source Data [file 44319_2025_611_MOESM11_ESM.zip › Figure EV3/Fig. EV3B/Fig. EV3B WT (cleaved caspase-3).jpg]

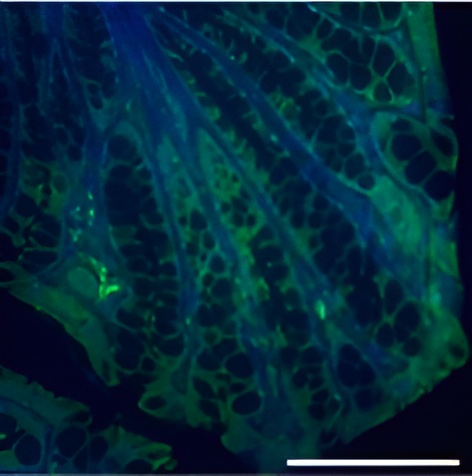

Supplement: Supplementary file 11 — Figure EV3 Source Data [file 44319_2025_611_MOESM11_ESM.zip › Figure EV3/Fig. EV3B/Fig. EV3B WT (cleaved caspase-3_DAPI).jpg]

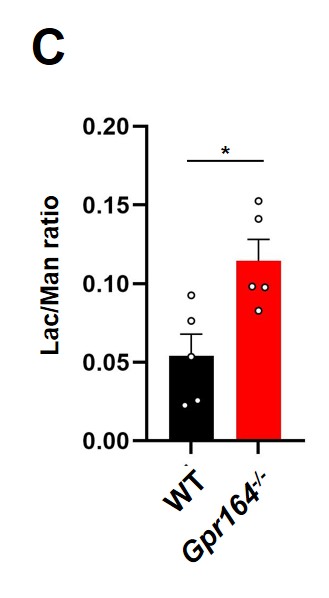

Supplement: Supplementary file 11 — Figure EV3 Source Data [file 44319_2025_611_MOESM11_ESM.zip › Figure EV3/Fig. EV3C/Fig. EV3C.jpg]

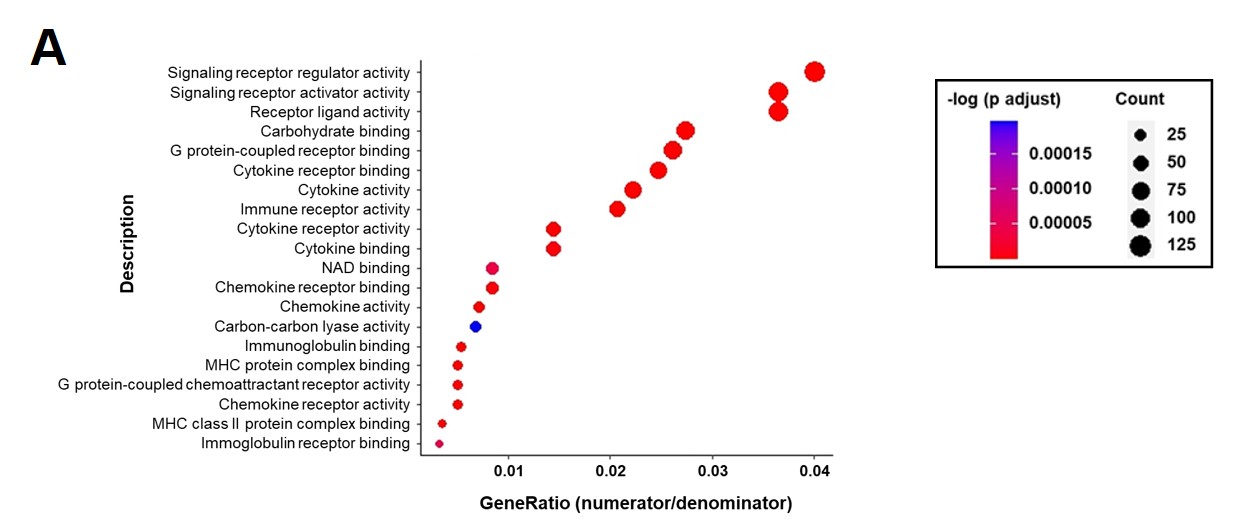

Supplement: Supplementary file 12 — Figure EV4 Source Data [file 44319_2025_611_MOESM12_ESM.zip › Figure EV4/Fig. EV4A/Fig. EV4A.jpg]

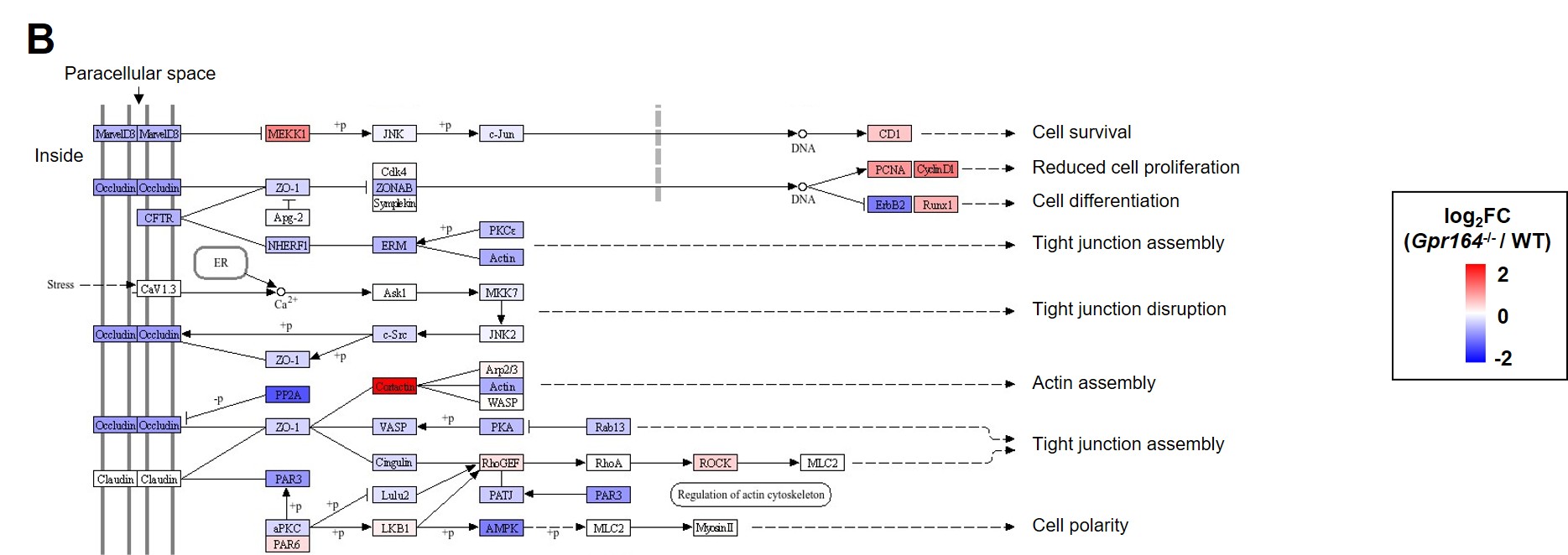

Supplement: Supplementary file 12 — Figure EV4 Source Data [file 44319_2025_611_MOESM12_ESM.zip › Figure EV4/Fig. EV4B/Fig. EV4B.jpg]

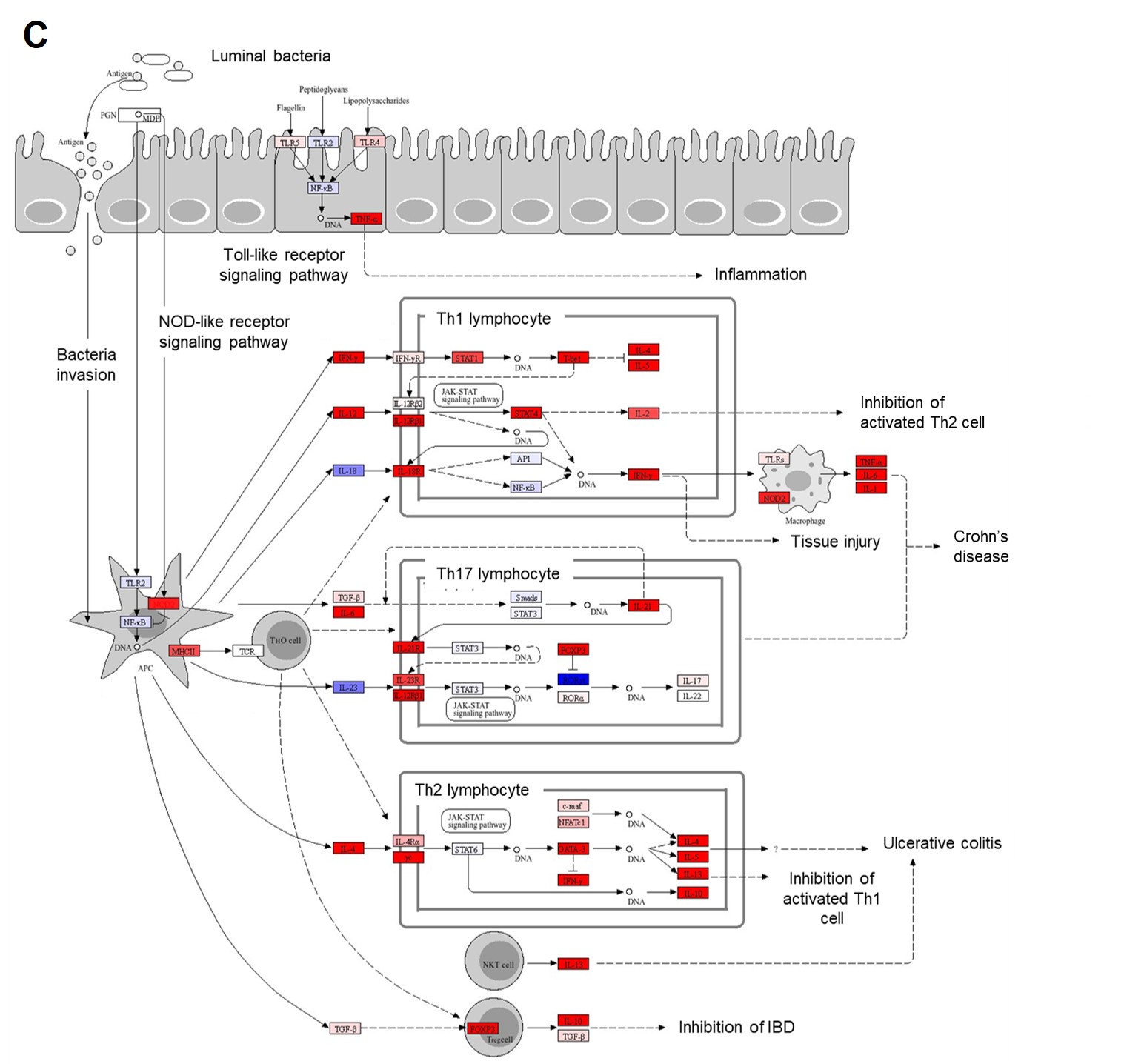

Supplement: Supplementary file 12 — Figure EV4 Source Data [file 44319_2025_611_MOESM12_ESM.zip › Figure EV4/Fig. EV4C/Fig. EV4C.jpg]

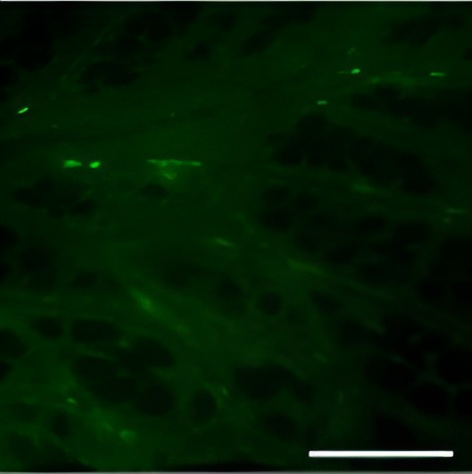

Supplement: Supplementary file 12 — Figure EV4 Source Data [file 44319_2025_611_MOESM12_ESM.zip › Figure EV4/Fig. EV4D/Fig. EV4D KO (CD4).jpg]

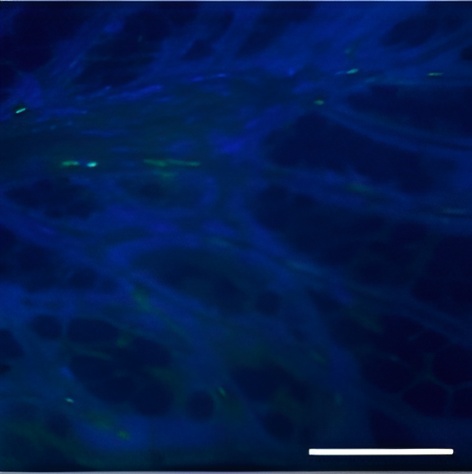

Supplement: Supplementary file 12 — Figure EV4 Source Data [file 44319_2025_611_MOESM12_ESM.zip › Figure EV4/Fig. EV4D/Fig. EV4D KO (CD4_DAPI).jpg]

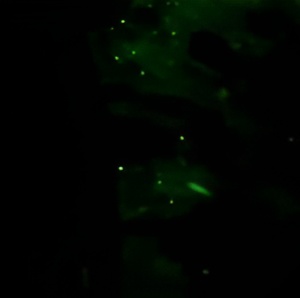

Supplement: Supplementary file 12 — Figure EV4 Source Data [file 44319_2025_611_MOESM12_ESM.zip › Figure EV4/Fig. EV4D/Fig. EV4D WT (CD4).jpg]

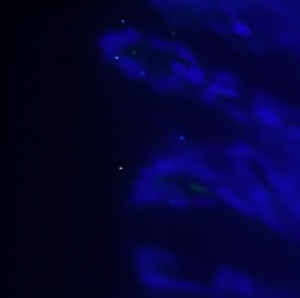

Supplement: Supplementary file 12 — Figure EV4 Source Data [file 44319_2025_611_MOESM12_ESM.zip › Figure EV4/Fig. EV4D/Fig. EV4D WT (CD4_DAPI).jpg]

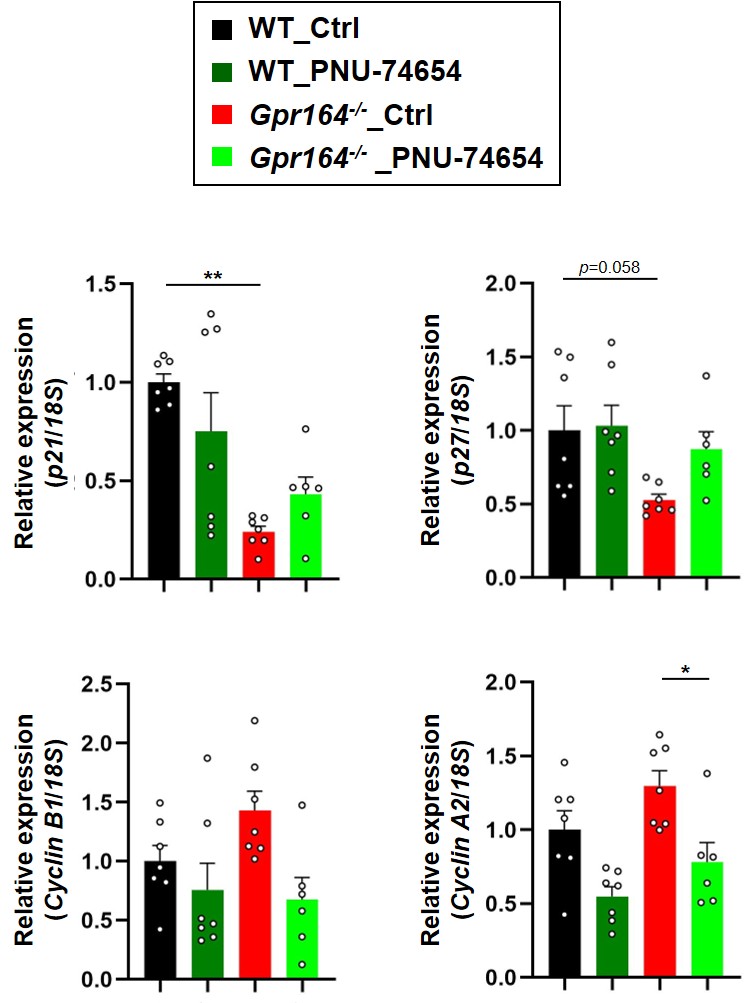

Supplement: Supplementary file 13 — Figure EV5 Source Data [file 44319_2025_611_MOESM13_ESM.zip › Figure EV5/Fig. EV5.jpg]
