# Supplementary material for: The free fatty acid receptor GPR164 maintains intestinal homeostasis and barrier function
Source: EMBO Rep. 2025 Oct 28;26(23):5905–30. doi: 10.1038/s44319-025-00611-5 (PMC12678807; doi:10.1038/s44319-025-00611-5)
Supplement: Supplementary file 14 — Expanded View Figures [file 44319_2025_611_MOESM14_ESM.pdf]

# Expanded View Figures

**Figure EV1. Generation of Or51e1-overexpressing cells, *OR51E1*-knockout cells, and *Or51e1*-knockout (*Gpr164*$^{-/-}$) mice.**

(**A**) Representative images of immunofluorescent staining for mouse Or51e1 in HEK293 cells. Cells were co-transfected with HA-tagged Or51e1 and receptor-transporting protein (left; RTP1S, right; RTP4), and stained with anti-HA antibody (green). DAPI was used for nuclei staining. Scale bar, 50 µm. (**B**) *OR51E1* expression in Caco-2 cells. *OR51E1*-deficient (*OR51E1* KO) cells were generated using the CRISPR/Cas9 system. For the detection of *OR51E1* expression, PCR amplification was done with the indicated primers, and the PCR products were separated on 1% agarose gels (left). The *OR51E1* mRNA expression level was determined by qRT-PCR ($n = 4$) (right). Error bars represent the mean ± SEM. **$P = 0.0021$ (Student's *t*-test). (**C**) Representative image of p53 protein expression. Cell extracts from Control KO or *OR51E1* KO cells were subjected to immunoblot analysis using an anti-p53 or anti-αTubulin antibody ($n = 3$). (**D**) Schematic representation of CRISPR/Cas9 targeting sites in *Or51e1* gene. *Or51e1* gene knockout (*Gpr164*$^{-/-}$) mice were generated by using the CRISPR/Cas9 system in wild-type C57BL/6J zygotes. Bold letters indicate the coding region of *Or51e1* gene. Red or Green letters indicate guide RNA (gRNA) and protospacer adjacent motif (PAM), respectively. (**E**) For the detection of the wild-type and mutant alleles, PCR amplification was done with the indicated primers, and the PCR products were separated on 1% agarose gels. (**F**) The *Or51e1* mRNA expression level in colon was determined by qRT-PCR ($n = 3$). Error bars represent the mean ± SEM. **$P = 0.0025$ (Student's *t*-test). Source data are available online for this figure.

© The Author(s)

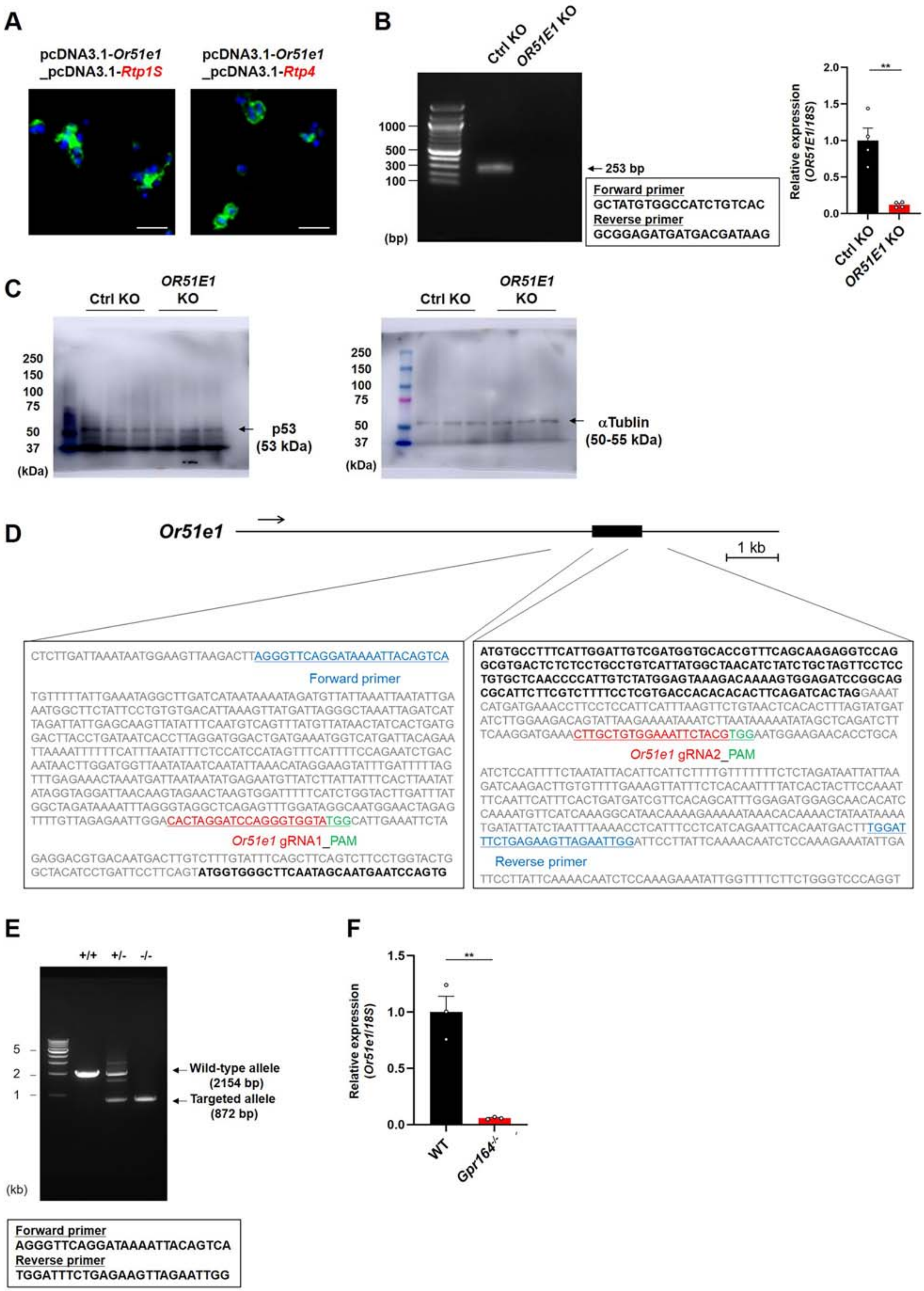
A
pcDNA3.1-Or51e1
_pcDNA3.1-Rtp1S
pcDNA3.1-Or51e1
_pcDNA3.1-Rtp4
B
Ctrl KO
OR51E1 KO
1000
500
300
100
(bp)
253 bp
Forward primer
GCTATGTGGCCATCTGTCAC
Reverse primer
GCGGAGATGATGACGATAAG
Relative expression (OR51E1/18S)
2.0
1.5
1.0
0.5
0.0
**
Ctrl KO
OR51E1 KO
C
Ctrl KO
OR51E1 KO
250
150
100
75
50
37
(kDa)
p53
(53 kDa)
Ctrl KO
OR51E1 KO
250
150
100
75
50
37
(kDa)
αTublin
(50-55 kDa)
D
Or51e1
1 kb
CTCTTGATTAAATAATGGAAGTTAAGACTTAGGGTTCAGGATAAAATTACAGTCA
Forward primer
TGTTTTTATTGAAATAGGCTTGATCATAATAAAATAGATGTTATTAAATTAATATTGA
AATGGCTTCTATTCCTGTGTGACATTAAAGTTATGATTAGGGCTAAATTAGATCAT
TAGATTATTGAGCAAGTTATATTTCAATGTCAGTTTATGTTATAACTATCACTGATG
GACTTACCTGATAATCACCTTAGGATGGACTGATGAAATGGTCATGATTACAGAA
TTAAAATTTTTTCATTTAATATTTCTCCATCCATAGTTTCATTTTCCAGAATCTGAC
AATAACTTGGATGGTTAATATAATCAATATTAAACATAGGAAGTATTTGATTTTTAG
TTTGAGAAACTAAATGATTAATAATATGAGAATGTTATCTTATTATTTCACTTAATAT
ATAGGTAGGATTAACAAGTAGAACTAAGTGGATTTTTCATCTGGTACTTGATTTAT
GGCTAGATAAAATTTAGGGTAGGCTCAGAGTTTGGATAGGCAATGGAACTAGAG
TTTTGTTAGAGAATTGGACACTAGGATCCAGGGTGGTATGGCATTGAAATTCTA
Or51e1 gRNA1_PAM
GAGGACGTGACAATGACTTGTCTTTGTATTTCAGCTTCAGTCTTCCTGGTACTG
GCTACATCCTGATTCCTTCAGTATGGTGGGCTTCAATAGCAATGAATCCAGTG
ATGTGCCTTTCATTGGATTGTCGATGGTGCACCGTTTCAGCAAGAGGTCCAG
GCGTGACTCTCTCCTGCCTGTCATTATGGCTAACATCTATCTGCTAGTTCCTCC
TGTGCTCAACCCCATTGTCTATGGAGTAAAGACAAAAGTGGAGATCCGGCAG
CGCATTCTTCGTCTTTTCCTCGTGACCACACACACTTCAGATCACTAGGAAAT
CATGATGAAACCTTCCTCCATTCATTTAAGTTCTGTAACTCACACTTTAGTATGAT
ATCTTGGAAGACAGTATTAAGAAAATAAATCTTAATAAAAATATAGCTCAGATCTT
TCAAGGATGAAACTTGCTGTGGAAATTCTACGTGGAATGGAAGAACACCTGCA
Or51e1 gRNA2_PAM
ATCTCCATTTTCTAATATTACATTCATTCTTTTGTTTTTTTCTCTAGATAATTATTAA
GATCAAGACTTGTGTTTTGAAAGTTATTTCTCACAATTTTATCACTACTTCCAAAT
TTCAATTCATTTCACTGATGATCGTTCACAGCATTTGGAGATGGAGCAACACATC
CAAAATGTTCATCAAAGGCATAACAAAAGAAAAATAAACACAAAACTATAATAAAA
TGATATTATCTAATTTAAAACCTCATTTCCTCATCAGAATTCACAATGACTTTGGAT
TTCTGAGAAGTTAGAATTGGATTCCTTATTCAAAACAATCTCCAAAGAAATATTGA
Reverse primer
TTCCTTATTCAAAACAATCTCCAAAGAAATATTGGTTTTCTTCTGGGTCCCAGGT
E
+/+
+/-
-/-
5
2
1
(kb)
Wild-type allele
(2154 bp)
Targeted allele
(872 bp)
Forward primer
AGGGTTCAGGATAAAATTACAGTCA
Reverse primer
TGGATTTCTGAGAAGTTAGAATTGG
F
Relative expression (Or51e1/18S)
1.5
1.0
0.5
0.0
**
WT
Gpr164-/-

 © The Author(s)

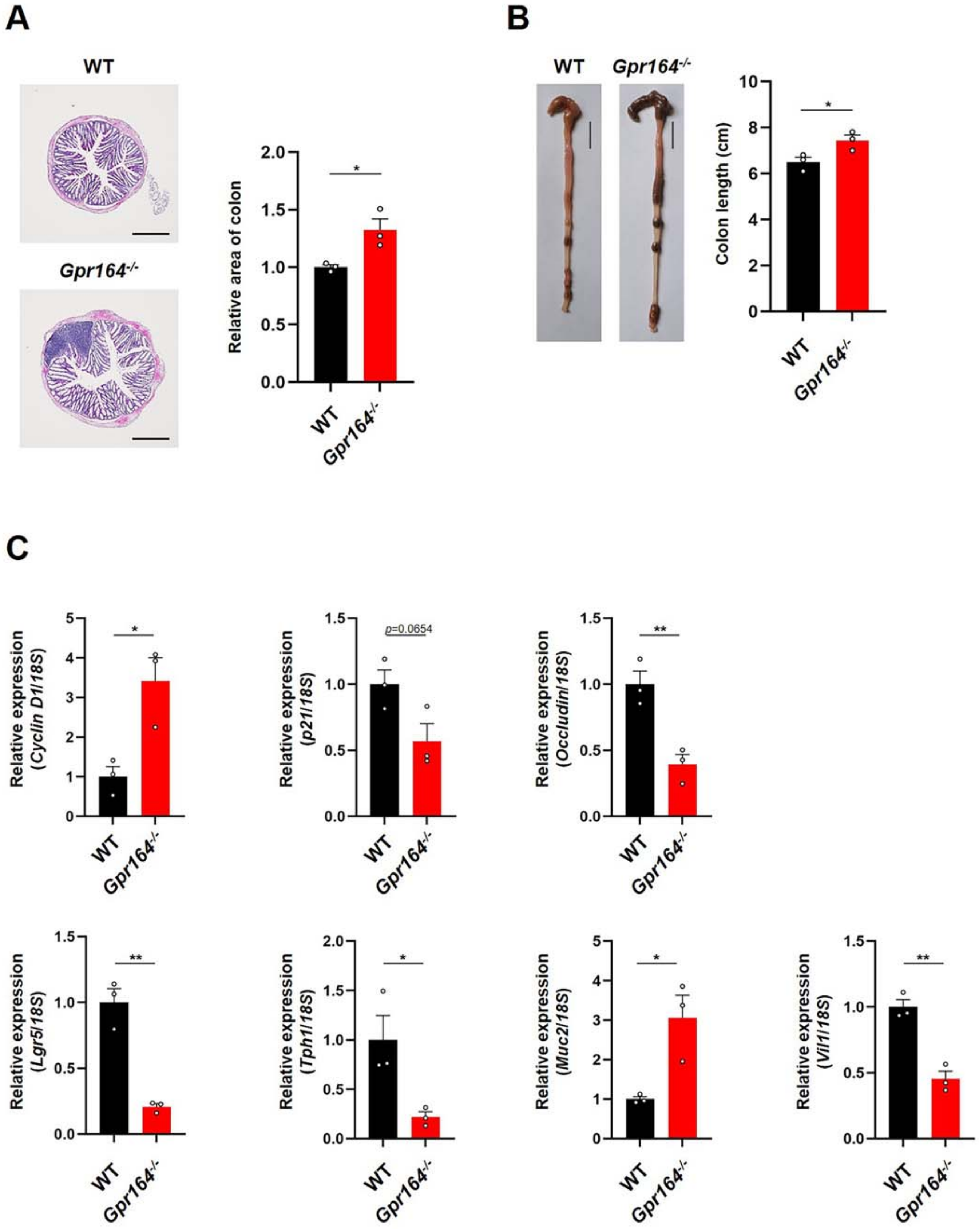
A
WT
Gpr164-/-
Relative area of colon
2.0
1.5
1.0
0.5
0.0
*
WT
Gpr164-/-
B
WT
Gpr164-/-
Colon length (cm)
10
8
6
4
2
0
*
C
Relative expression (Cyclin D1/18S)
*
Relative expression (p21/18S)
p=0.0654
Relative expression (Occludin/18S)
**
Relative expression (Lgr5/18S)
**
Relative expression (Tph1/18S)
*
Relative expression (Muc2/18S)
*
Relative expression (Vil1/18S)
**

**Figure EV2. Phenotypes of colonic hyperplasia in female *Gpr164*$^{-/-}$ mice.**

(**A**) Representative images of colon sections stained with hematoxylin-eosin (HE). The cross-sections of colon obtained from female WT and female *Gpr164*$^{-/-}$ mice were stained with HE, and the area of colon was measured using ImageJ software ($n = 3$). Scale bar, 500 µm. Error bars represent the mean ± SEM. $^{*}P = 0.029$ (Student's *t*-test). (**B**) Representative images of colon ($n = 3$). Scale bar, 1 cm. Error bars represent the mean ± SEM. $^{*}P = 0.0405$ (Student's *t*-test). (**C**) The expression levels of genes related to cell cycle, intestinal barrier and epithelial lineage were determined by qRT-PCR. Total RNA was extracted from colon tissue of female WT and female *Gpr164*$^{-/-}$ mice ($n = 3$). Error bars represent the mean ± SEM. $^{*}P = 0.0194$; *Cyclin D1* gene, $P = 0.0654$; *p21* gene, $^{**}P = 0.0083$; *Occludin* gene, $^{**}P = 0.0018$; *Lgr5* gene, $^{*}P = 0.0366$; *Tph1* gene, $^{*}P = 0.023$; *Muc2* gene, $^{**}P = 0.0024$; *Vil1* gene (*Tph-1* gene; Mann–Whitney *U*-test, other genes; Student's *t*-test). Source data are available online for this figure.

 © The Author(s)

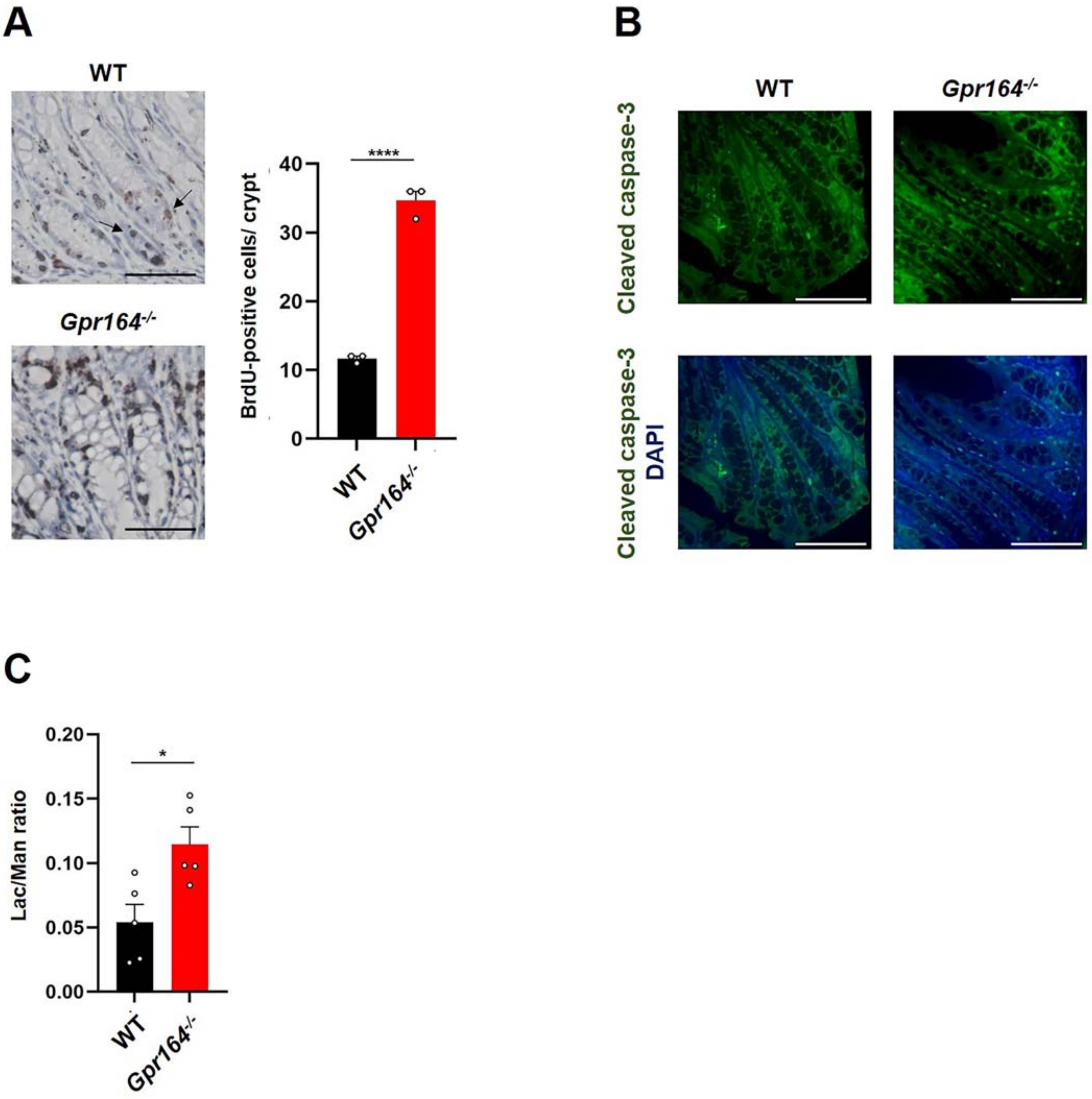


**Figure EV3. Effects of *Gpr164* deletion on proliferation, apoptosis, and intestinal barrier function.**

(**A**) Representative images of 5-bromo-2′-deoxyuridine (BrdU)-labeled cells in colon. The cross-sections of colon obtained from WT and *Gpr164*$^{-/-}$ mice were stained with anti- BrdU antibody, and visualized with DAB ($n = 3$). Hematoxylin was used for nuclei staining. Scale bar, 50 µm. Error bars represent the mean ± SEM. ****$P < 0.0001$ (Student's *t*-test). (**B**) Representative images of immunofluorescent staining for cleaved caspase-3. The cross-sections of colon obtained from WT and *Gpr164*$^{-/-}$ mice were stained with anti-cleaved caspase-3 antibody ($n = 3$). DAPI was used for nuclei staining. Scale bar, 50 µm. (**C**) Assessment of intestinal permeability by measuring urinary lactulose and mannitol. The excretion ratio of lactulose/mannitol in urine was assessed ($n = 5$). Error bars represent the mean ± SEM. *$P = 0.0143$ (Student's *t*-test). Source data are available online for this figure.

© The Author(s)

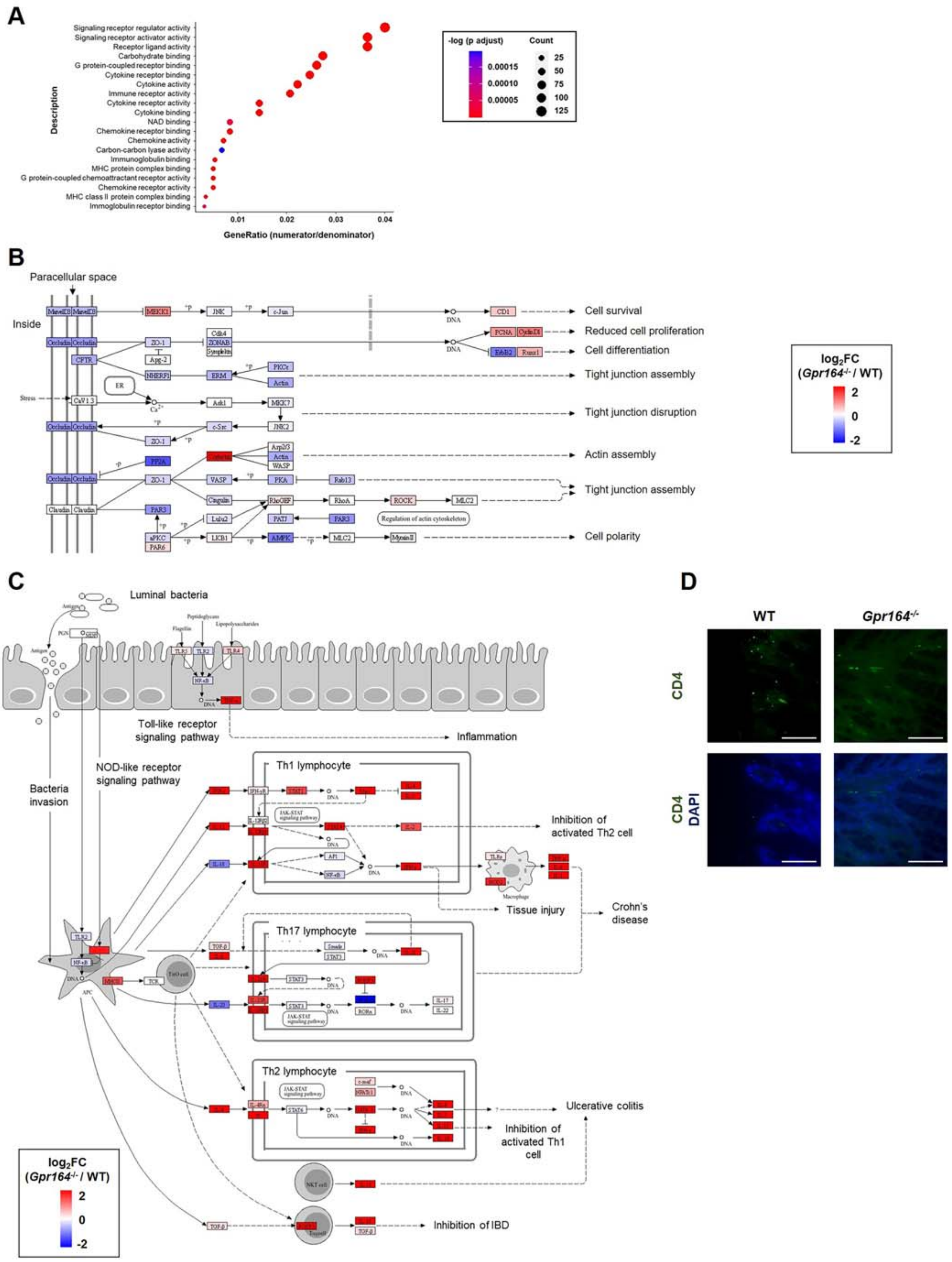
A
Signaling receptor regulator activity
Signaling receptor activator activity
Receptor ligand activity
Carbohydrate binding
G protein-coupled receptor binding
Cytokine receptor binding
Cytokine activity
Immune receptor activity
Cytokine receptor activity
Cytokine binding
NAD binding
Chemokine receptor binding
Chemokine activity
Carbon-carbon lyase activity
Immunoglobulin binding
MHC protein complex binding
G protein-coupled chemoattractant receptor activity
Chemokine receptor activity
MHC class II protein complex binding
Immoglobulin receptor binding
Description
0.01
0.02
0.03
0.04
GeneRatio (numerator/denominator)
-log (p adjust)
0.00015
0.00010
0.00005
Count
25
50
75
100
125
B
Paracellular space
Inside
Cell survival
Reduced cell proliferation
Cell differentiation
Tight junction assembly
Tight junction disruption
Actin assembly
Tight junction assembly
Cell polarity
log2FC
(Gpr164-/- / WT)
2
0
-2
C
Luminal bacteria
Toll-like receptor signaling pathway
Inflammation
NOD-like receptor signaling pathway
Bacteria invasion
Th1 lymphocyte
Inhibition of activated Th2 cell
Tissue injury
Crohn's disease
Th17 lymphocyte
Th2 lymphocyte
Ulcerative colitis
Inhibition of activated Th1 cell
Inhibition of IBD
log2FC
(Gpr164-/- / WT)
2
0
-2
D
WT
Gpr164-/-
CD4
CD4
DAPI

 © The Author(s)

**Figure EV4. Genome-wide RNA sequencing of *Gpr164*$^{-/-}$ mice.**

(**A**) KEGG enrichment analysis involved in the molecular function in colon of *Gpr164*$^{-/-}$ mice ($n = 5$). *P* values were adjusted based on the false discovery rate (FDR). (**B**, **C**) KEGG pathway enrichment related to tight junction (**B**) and inflammatory bowel disease (**C**). Increased or decreased levels of gene expressions are shown in red or blue, respectively. (**D**) Representative images of immunofluorescent staining for CD4. The cross-sections of colon obtained from WT and *Gpr164*$^{-/-}$ mice were stained with anti-CD4 antibody ($n = 3$). DAPI was used for nuclei staining. Scale bar, 50 µm. Source data are available online for this figure.

© The Author(s)

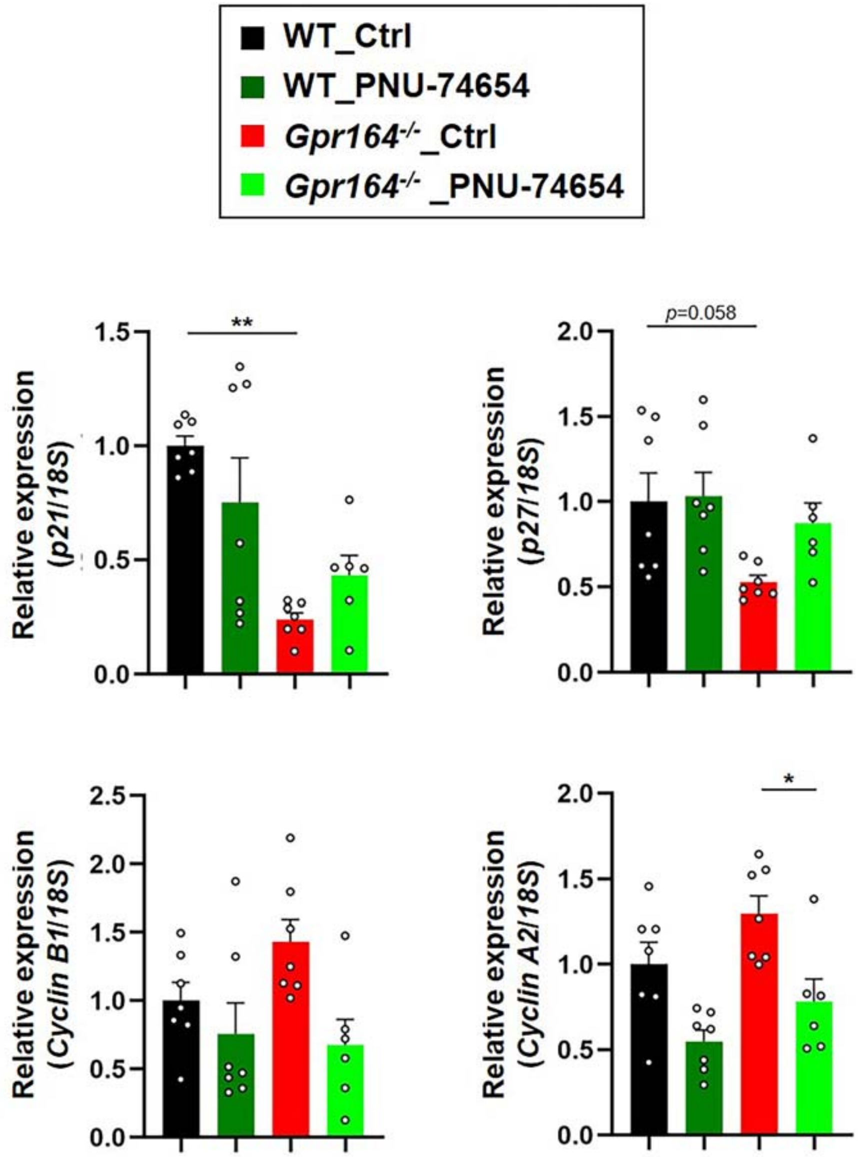


**Figure EV5. Cell cycle gene expressions in PNU-74654-treated *Gpr164*$^{-/-}$ mice.**

The mRNA expression levels of cell cycle genes determined by qRT-PCR ($n = 6$–7). WT and *Gpr164*$^{-/-}$ mice were injected intraperitoneally with PNU-74654 (15 mg/kg body weight, every 2 days for 3 weeks), and total RNA was extracted from colon tissue of WT and *Gpr164*$^{-/-}$ mice ($n = 6$–7). Error bars represent the mean ± SEM. **$P = 0.0028$; WT_Ctrl vs *Gpr164*$^{-/-}$_Ctrl of *p21* gene, $P = 0.0586$; WT_Ctrl vs *Gpr164*$^{-/-}$_Ctrl of *p27* gene, *$P = 0.0173$; *Gpr164*$^{-/-}$_Ctrl vs *Gpr164*$^{-/-}$_ PNU-74654 of *Cyclin A2* gene (*p21* and *Cyclin B1* genes; Dunn's test, *p27* and *Cyclin A2* genes; Tukey–Kramer test). Source data are available online for this figure.

 © The Author(s)
